# Synthesis of fucosyllactose using α-L-fucosidases GH29 from infant gut microbial metagenome

**DOI:** 10.1007/s00253-024-13178-3

**Published:** 2024-05-21

**Authors:** Eva M. Moya-Gonzálvez, Birgitte Zeuner, Albert Th. Thorhallsson, Jesper Holck, Martina Palomino-Schätzlein, Jesús Rodríguez-Díaz, Anne S. Meyer, María J. Yebra

**Affiliations:** 1 Laboratorio de Bacterias Lácticas y Probióticos, Departamento de Biotecnología de Alimentos, Instituto de Agroquímica y Tecnología de Alimentos (IATA-CSIC), Valencia, Spain; 2 Protein Chemistry and Enzyme Technology, Department of Biotechnology and Biomedicine, Technical University of Denmark, Lyngby, Denmark; 3 ProtoQSAR, CEEI, Parque Tecnológico Valencia, 46980 Paterna, Spain; 4 Departamento de Microbiología, Facultad de Medicina, Universidad de Valencia, Valencia, Spain

**Keywords:** α-L-fucosidase, GH29, Human milk oligosaccharides, 2′-fucosyllactose, 3′-fucosyllactose, Protein engineering

## Abstract

**Abstract:**

Fucosyl-oligosaccharides (FUS) provide many health benefits to breastfed infants, but they are almost completely absent from bovine milk, which is the basis of infant formula. Therefore, there is a growing interest in the development of enzymatic transfucosylation strategies for the production of FUS. In this work, the α-L-fucosidases Fuc2358 and Fuc5372, previously isolated from the intestinal bacterial metagenome of breastfed infants, were used to synthesize fucosyllactose (FL) by transfucosylation reactions using *p*-nitrophenyl-α-L-fucopyranoside (pNP-Fuc) as donor and lactose as acceptor. Fuc2358 efficiently synthesized the major fucosylated human milk oligosaccharide (HMO) 2′-fucosyllactose (2′FL) with a 35% yield. Fuc2358 also produced the non-HMO FL isomer 3′-fucosyllactose (3′FL) and traces of non-reducing 1-fucosyllactose (1FL). Fuc5372 showed a lower transfucosylation activity compared to Fuc2358, producing several FL isomers, including 2′FL, 3′FL, and 1FL, with a higher proportion of 3′FL. Site-directed mutagenesis using rational design was performed to increase FUS yields in both α-L-fucosidases, based on structural models and sequence identity analysis. Mutants Fuc2358-F184H, Fuc2358-K286R, and Fuc5372-R230K showed a significantly higher ratio between 2′FL yields and hydrolyzed pNP-Fuc than their respective wild-type enzymes after 4 h of transfucosylation. The results with the Fuc2358-F184W and Fuc5372-W151F mutants showed that the residues F184 of Fuc2358 and W151 of Fuc5372 could have an effect on transfucosylation regioselectivity. Interestingly, phenylalanine increases the selectivity for α-1,2 linkages and tryptophan for α-1,3 linkages. These results give insight into the functionality of the active site amino acids in the transfucosylation activity of the GH29 α-L-fucosidases Fuc2358 and Fuc5372.

**Key points:**

- *Two α-L-fucosidases from infant gut bacterial microbiomes can fucosylate glycans*
- *Transfucosylation efficacy improved by tailored point-mutations in the active site*
- *F184 of Fuc2358 and W151 of Fuc5372 seem to steer transglycosylation regioselectivity*

**Supplementary Information:**

The online version contains supplementary material available at 10.1007/s00253-024-13178-3.

## Introduction

Human milk oligosaccharides (HMOs) are present in breast milk at concentrations ranging from 5 to 25 g l^−1^, which vary during the course of lactation and between women. They are the third most abundant solid component of human milk after lipids and lactose (Kunz et al. 2000). Between 50 and 80% of the HMOs present in human milk are fucosyl-oligosaccharides (FUS) (Gabrielli et al. 2011), although the concentration can vary according to the secretor and Lewis blood status group of the mother, and also during lactation (Kunz et al. 2017). Among all the FUS, 2′-fucosyllactose (2′FL) is the most abundant fucosylated HMO in secretor mothers, constituting between 15 and 30% of the total amount of HMOs (Thurl et al. 2017). Other FUS are present in high amounts in human milk from secretor mothers, such as 3-fucosyllactose (3FL), lacto-*N*-difucohexaose I (LNDFH I), and lacto-*N*-fucopentaose I (LNFP I); and in non-secretor mothers, such as lacto-*N*-fucopentaose II (LNFP II), lacto-*N*-fucopentaose III (LNFP III), and lacto-*N*-difucohexaose II (LNDFH II) (Walsh et al. 2020).

The beneficial effects of HMOs in breastfed infants have been widely described (Bode 2012). Particularly, FUS can act as antiadhesive antimicrobials, preventing bacterial and viral infections (Gozalbo-Rovira et al. 2019; Ray et al. 2019). In addition, some intestinal bacteria in breastfed infants are able to metabolize FUS, promoting the growth and shaping a gut microbiota with health benefits for infants (Sakanaka et al. 2019; Zuñiga et al. 2018; Zuñiga et al. 2020). Hydrolysis of FUS has been described by some intestinal bacteria belonging to *Bifidobacterium* and *Lactobacillus* genera, which encode α-L-fucosidases responsible for the release of fucose moieties (Rodriguez-Diaz et al. 2011; Sela et al. 2012). The utilization of FUS by some *Enterococcus* and *Streptococcus* strains isolated from the intestinal microbiota has been reported (Yu et al. 2013), although no α-L-fucosidases have been characterized from these organisms. Less than 1% of the oligosaccharides present in bovine milk, which is used to make infant formula, are fucosylated (Aldredge et al. 2013). For this reason, the production of synthetic FUS is of great interest in order to obtain infant formulas that are more similar to human milk, thus ensuring their beneficial effects on infants (Thurl et al. 2017).

Chemical synthesis has been used to produce HMOs (Kameyama et al. 1991), but it is a lengthy and expensive process due to the multiple protection and deprotection steps needed to achieve adequate selectivity. Enzymatic synthesis is an alternative that, despite the presence of other functional groups, is easier and rather economically affordable due to its higher glycosidic linkage specificity. Glycosyltransferases (GTs) and glycoside hydrolases (GHs) can carry out reactions to synthesize HMOs. GTs have high specificity toward the acceptor and do not hydrolyze the product, but they are difficult to express and usually require complex multistep enzymatic systems for nucleotide substrate regeneration (Nidetzky et al. 2018). In contrast, GHs are easier to produce and, although they have less specificity than GTs, they have been widely used for transglycosylation synthesis due to the flexibility to use different donor and acceptor substrates. GHs function with either an inverting or retaining catalytic mechanism. The former uses a single-step mechanism in which the leaving group is directly displaced by the nucleophilic water molecule, giving to the product an inverted anomeric configuration. The retaining mechanism involves two catalytic carboxylates, an acid-base and a nucleophile, and consists of two steps, the formation of a covalent glycosyl enzyme intermediate and its cleavage in the presence of a water molecule acting as a nucleophile. When an acceptor different from water, such as an alcohol or a sugar, intercepts the reactive intermediate, the retaining glycosidases work as transglycosylases with the capacity to synthesize carbohydrates (Bojarová and Křen 2009). Inverting α-L-fucosidases are members of CAZy family GH95, while most retaining α-L-fucosidases belong to GH29 (www.cazy.org). This family has been further divided into the subfamilies GH29A and GH29B, according to sequence homology and substrate specificity (Sakurama et al. 2012b). GH29A contains enzymes with relatively relaxed substrate specificities, whereas GH29B are more specific for α-1,3/4 linkages. FUS have been previously obtained by transfucosylation reaction using different α-L-fucosidases (Guzman-Rodriguez et al. 2018; Rodriguez-Diaz et al. 2013; Zeuner and Meyer 2020; Zeuner et al. 2018a). The main disadvantage of GHs for FUS production is their moderate transglycosylation activity compared to their hydrolysis activity, resulting in low product yields. Different approaches have been taken in order to increase the efficiency of the transglycosylation reactions (Zeuner and Meyer 2020; Zeuner et al. 2019), and protein engineering has been widely used to increase the transglycosylation activity of various GHs, including α-L-fucosidases (Saumonneau et al. 2016; Teze et al. 2021; Zeuner et al. 2018b).

We have previously identified and characterized several α-L-fucosidases by metagenomic analysis of the intestinal microbiota of breastfed infants (Moya-Gonzalvez et al. 2022). These enzymes, belonging to the GH29 family, showed different substrate specificities toward fucosylated HMOs, histo-blood group antigens, and glycoproteins. In this work, the transglycosylation activity of the α-L-fucosidases Fuc2358 and Fuc5372 was evaluated. Both enzymes were subjected to rational design in order to increase their transglycosylation efficiency. The rational selection of mutations was based on previous studies of mutant α-L-fucosidases with high transfucosylation activity, as well as sequence and three-dimensional structural alignments.

## Materials and methods

### Transfucosylation activity assay

The α-L-fucosidases Fuc19A, Fuc35A, Fuc35B, Fuc1584, Fuc2358, and Fuc5372 (GenBank accession numbers: Fuc19A, ON170362; Fuc35A, ON170364; Fuc35B, ON170365; Fuc1584, ON170368; Fuc2358, ON170369; Fuc5372, ON170370) were expressed with a 6xHis-tag and purified as previously described (Moya-Gonzalvez et al. 2022). The transfucosylation activity of the 6xHis-tagged α-L-fucosidases was assessed in a solution containing 100 mM NaH_2_PO_4_/Na_2_HPO_4_ buffer, pH 6.0 or 6.5, based on the optimal pH of each enzyme (Moya-Gonzalvez et al. 2022), *p*-nitrophenyl-α-L-fucopyranoside (pNP-Fuc) 50 mM as a donor, and lactose (Lac) 150 mM as acceptor. Samples were heated at 100 °C during 10 min for pNP-Fuc to solubilize and then cooled down to the reaction temperature (40 or 45 °C). Reactions were started by adding 10 μg (50 μg ml^−1^) of enzyme and samples were withdrawn at different times and immediately heated at 100 °C for 10 min to stop the reaction.

Transfucosylation reactions (200 μL) aimed at the purification of the produced fucosyllactose (FL) trisaccharides were carried out under the conditions described above for 4 h, followed by heat treatment at 100 °C for 10 min. Reactions of Fuc2358 and Fuc5372 were concentrated with a rotary evaporator to a final volume of 20 μL and 40 μL, respectively.

### High-performance liquid chromatography (HPLC) analysis

Transfucosylation reaction products were analyzed by high-performance liquid chromatography (HPLC) with a Jasco PU2080Plus system coupled to a refractive index detector (Jasco RI-2031 Plus) using an ion-exclusion chromatography column (Rezex RSO-Oligosaccharide Ag+ column; Phenomenex, Torrance, CA, USA). The column was kept at 80 °C and the samples were eluted in an isocratic mode with water as mobile phase, at a flow rate of 0.3 mL min^−1^. The FUS synthesized were confirmed by comparison of their retention times with those of standards 2′FL, 3FL, and 2′,3-difucosyllactose (DFL) (Biosynth AG, Staad, Switzerland). To purify the synthesized oligosaccharides, the corresponding fractions from the Fuc2358 and Fuc5372 transglycosylation reactions containing FL were pooled and concentrated using a rotary evaporator and named as F1 and F2, respectively. The synthesized oligosaccharides contained in fractions F1 and F2 were confirmed by comparison of their retention times with those of standards 2′FL, 3FL, DFL, and 6′-fucosyllactose (6′FL) (Biosynth AG, Staad, Switzerland) by high-pH anion-exchange chromatography with pulsed amperometric detection (HPAEC-PAD) in a Dionex ICS3000 system using a CarboPac™PA100 analytical column equipped with a CarboPac™PA100 guard column (Dionex Corp., Sunnyvale, CA, USA). A gradient of 10 to 100 mM NaOH in 30 min with a flow rate of 1 mL min′^1^ was used at 27 °C.

### Nuclear magnetic resonance spectroscopy (NMR) analysis

Lyophilized samples were dissolved in 550 μL of deuterated water and stored at − 80 °C until analysis. Nuclear magnetic resonance (NMR) spectra were recorded at 25 °C using a Bruker Avance II 600 MHz spectrometer (Bruker Daltonics, Bremen, Germany) equipped with a 5-mm TCI cryoprobe. One-dimensional (1D) ^1^H spectra with presaturation and a noisy mixing period for water suppression and improved baseline were acquired with 256 transients. ^1^H heteronuclear single quantum coherence (HSQC) experiments were acquired with 200 transients over a spectral width of 3.5 (for ^1^H) and 128 points in the indirect dimension. Total correlation spectroscopy (TOCSY) experiments were acquired with 32 transients over a spectral width of 3.5 ppm in both dimensions and 128 points in the indirect dimension. NMR spectra were processed using the program Topspin3.2 (Bruker Biospin).

Characterization of 3′-fucosyllactose (3′FL): ^1^HNMR (CDCl3, 600 MHz) δH: 5.21 (d, 1H, J 2.0 Hz, H-1), 5.16 (d, 2H, J 4.0 Hz, H-1′ and H-1″), 4,66 (d, 1H, J 8.0 Hz, H-2), 4,51 (d, 2H, J 6.0 Hz, H-2′ and H-2″), 4,02-3,51 (m, 8H, H4–H6, H4′ ,–H6′ , H3″–H4″), 1,20 (d, 3H, J 7.0 Hz, H-6″).

### Liquid chromatography electrospray ionization mass spectrometry (LC-ESI-MS) analysis

Transfucosylation reaction products were analyzed by liquid chromatography electrospray ionization mass spectrometry (LC-ESI-MS) on an Amazon SL Iontrap (Bruker Daltonics, Bremen, Germany) coupled to an UltiMate 3000 UHPLC from Dionex (Sunnyvale, CA, USA) equipped with a porous graphitized carbon column (Hypercarb PGC, 150 mm × 2.1 mm, 3 μm; Thermo Fisher Scientific, Waltham, MA, USA). Ten or 30 μL of 4 h transfucosylation reaction sample was injected and analyzed in negative mode as described previously (Zeuner et al. 2018a), using a target mass of 550 m/z. Products were observed as deprotonated [M-H]^−^ and formate adducts [M-H + FA]^−^. Identification based on m/z values and MS^2^ fragmentation was performed in Compass Data Analysis 5.3 (Bruker Daltonics).

For fractionation of individual isomers, 100 μL of 4 h transfucosylation reaction sample was injected and collected by hand based on retention time, dried in a speed vacuum concentrator, and resolubilized in miliQ water, as previously described (Vuillemin et al. 2021). For structural elucidation of the products, selected reaction samples were reduced according to Vuillemin et al. with some modifications: selected transfucosylation fractions were mixed with freshly made aqueous NaBH_4_ to a final concentration of 0.125 M and incubated for 15 h at room temperature (Vuillemin et al. 2021). Reactions were stopped by adding 0.085 M of acetic acid. The fractions were also treated with 1 U of β-galactosidase from *Aspergillus oryzae* (Sigma-Aldrich, Steinheim, Germany) as previously described (Nordvang et al. 2016), and the reactions were stopped by heating at 100 °C after overnight incubation at 30 °C.

### Structural models and molecular docking

Structural models for the enzymes studied here, Fuc2358 and Fuc5372, and for AlfB from *Lacticaseibacillus paracasei* (ex-*Lactobacillus casei*) were obtained using AlphaFold2 (version 1.3.0) (Mirdita et al. 2022) and ranked, selecting the model with the lowest energy for each enzyme. The active site of Fuc2358 and Fuc5372 was established with Python Molecular Viewer (PMV, version 1.5.7) and AutoDock Vina software (version 1.5.7) was used for lactose docking in the Fuc2358 structure. α-L-fucose extracted from the crystal structure of the α-L-fucosidase E1_10125 mutant from *Ruminococcus gnavus* (PDB 6TR4) was superimposed on Fuc2358 for the analysis of its retaining mechanism. For mutant selection, structural and sequence alignments were performed. Multiple sequence alignment (MSA) of characterized α-L-fucosidases belonging to the CAZy family GH29 together with α-L-fucosidases previously isolated from the intestinal microbiota of breastfed infants (Moya-Gonzalvez et al. 2022) was performed using Clustal Omega (Sievers et al. 2011). Fuc2358 and Fuc5372 AlphaFold2 structures were structurally aligned to each other as well as to the AlphaFold2 structure of AlfB from *Lacticaseibacillus paracasei* using PyMOL (version 2.5.4). All the 3D structures shown are represented with PyMOL.

### Cloning, expression, and purification of mutant α-L-fucosidases

All mutants were constructed using CloneAmp^TM^ polymerase (Takara, Kusatsu, Japan), a set of mutagenic primers (Table S1), and vector pQEfuc2358 or pQEfuc5372 (Moya-Gonzalvez et al. 2022) as template. The PCR products were purified using the Gel Band Purification Kit (GE HealthCare Life Sciences, Chicago, IL, USA). The amplified recombinant genes and plasmid pQE80L (Qiagen) were digested with the same pair of enzymes (SphI/HindIII for Fuc2358 and BamHI/HindIII for Fuc5372) and ligated with T4 DNA ligase (Thermo Fisher Scientific, Waltham, MA, USA). *Escherichia coli* DH10B was transformed by thermal shocking at 42 °C for 45 s and different dilutions were incubated in Luria-Bertani (LB) agar plates with ampicillin 100 μg mL^−1^ at 37 °C overnight. One clone of each was selected, E159, E160, E161, E162, E163, E164, E165, E166, E167, and E168 (Table S2), and the corresponding plasmids were extracted using GeneJET Plasmid Miniprep Kit (Thermo Fisher Scientific, Waltham, MA, USA). All constructs were checked by DNA sequencing to confirm the correct sequence of the inserts (Macrogen Europe, Amsterdam, The Netherlands). Expression and purification of recombinant α-L-fucosidases were carried out in *E. coli* DH10B, as described previously (Moya-Gonzalvez et al. 2022). Purified proteins were confirmed by SDS-PAGE and protein assay dye reagent concentrate (BioRad, Hercules, California, USA) was used to determine the protein concentration.

### Hydrolytic and transfucosylation activity of mutant α-L-fucosidases

One hundred microliters of reaction mixtures containing 5 mM of pNP-Fuc and 100 mM of Tris-HCl buffer pH 7.0 were initiated by adding 1 μg of enzyme. The hydrolytic activity was determined by measuring the *p*-nitrophenol released from pNP-Fuc at 37 °C for 15 min at 404 nm in a spectrophotometer (POLARstar Omega microplate reader, BMG Labtech), as previously described (Moya-Gonzalvez et al. 2022). Reactions without enzyme were included as negative controls. The hydrolytic activity of the mutant enzymes was defined as a relative percentage of the activity of their respective wild type (WT).

Transfucosylation activity of mutant α-L-fucosidases was monitored as described above by adding 50 μg mL^−1^ or 500 μg mL^−1^ of the enzyme to the reaction mixture. Analysis of the products was carried out by HPAEC-PAD as described above.

### Statistical analysis

One-way ANOVA followed by Tukey’s HSD test for multiple comparisons for determination of statistical significance was performed using GraphPad Prism, version 8.4.3 (GraphPad Software Inc., San Diego, CA, USA). Significant differences (*p* < 0.05) were indicated with superscript letters (a–e) for each parameter between variants of the same enzyme.

## Results

### Synthesis, purification, and identification of fucosyllactose oligosaccharides

**Fig. 1:**
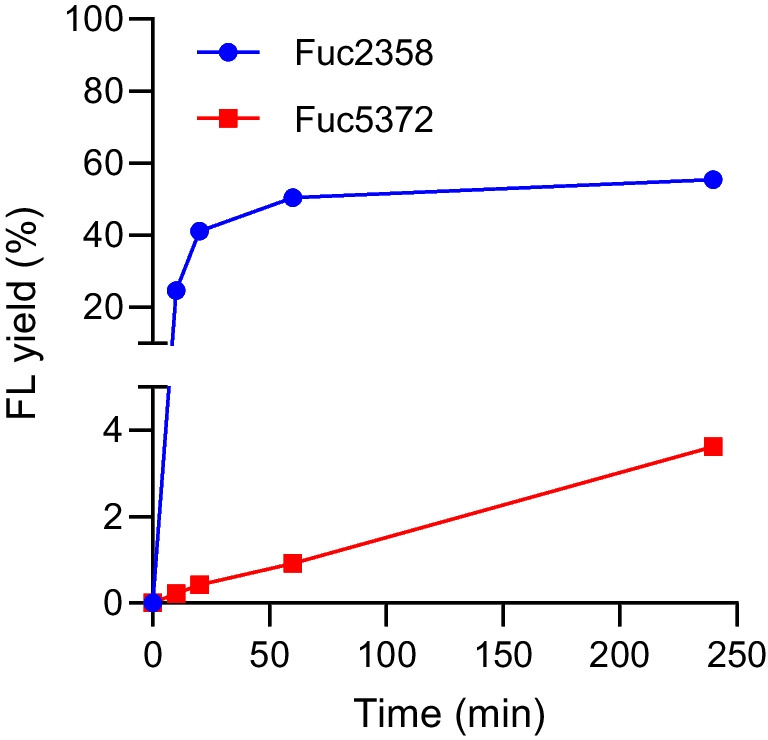
Time-dependent synthesis of fucosyllactose (FL) by transfucosylation reactions catalyzed by the α-L-fucosidases Fuc2358 (blue circles) and Fuc5372 (red squares) and analyzed by HPLC-Jasco system. Yields are expressed as a percentage of the donor concentration (*p*-nitrophenyl-α-L-fucopyranoside 50 mM)

**Fig. 2:**
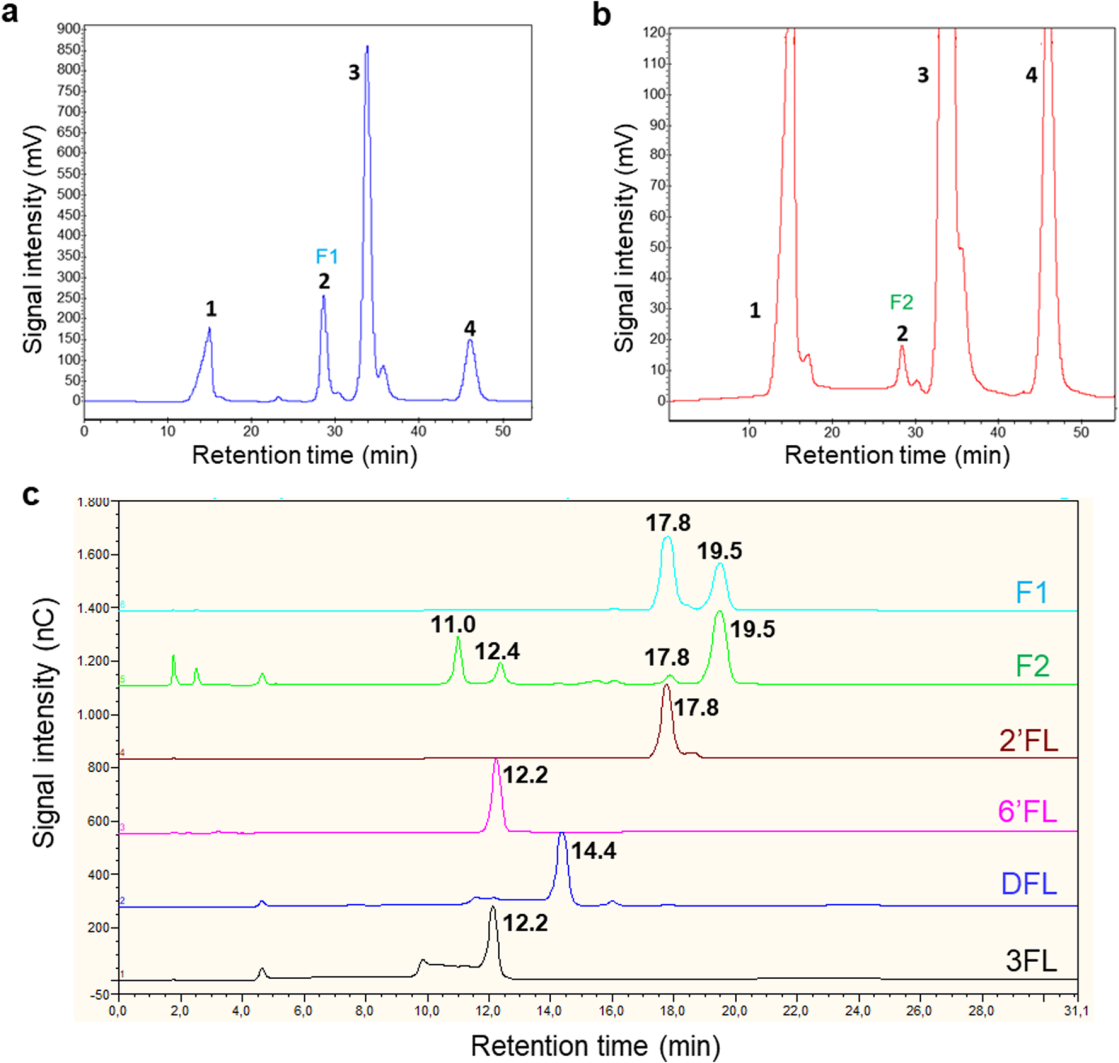
HPLC chromatograms (Jasco PU2080Plus coupled to a refractive index detector) of Fuc2358 (**a**) and Fuc5372 (**b**) transfucosylation reactions, 1, Tris-HCl; 2, F1 or F2 fraction containing fucosyllactose (FL) with the same retention time as the 2′-fucosyllactose (2′FL) standard; 3, lactose; 4, mixture of L-fucose and glycerol. HPAEC-PAD chromatograms (Dionex ICS3000 system) of the purified fractions F1 from Fuc2358 and F2 from Fuc5372 (**c**), and 2′FL, 6′fucosyllactose (6’FL), 2′,3-difucosyllactose (DFL), and 3-fucosyllactose (3FL) standards. The numbers indicate the retention times of the peaks in minutes

Six α-L-fucosidases (Fuc19A, Fuc35A, Fuc35B, Fuc1584, Fuc2358, and Fuc5372), previously isolated and characterized from the intestinal microbiota of breastfed infants (Moya-Gonzalvez et al. 2022), were selected for this study based on their hydrolytic activity towards 2′FL (Fucα1-2Galβ1-4Glc) and/or 3FL (Galβ1-4(Fucα1-3)Glc) and their ability to hydrolyze the artificial substrate pNP-Fuc. Transfucosylation reactions with those α-L-fucosidases were performed using pNP-Fuc as donor substrate and lactose as acceptor substrate. Only the α-L-fucosidases Fuc2358 and Fuc5372 produced fucosyllactose (FL) (Fig. 1) and the product retention time was the same as the 2′FL standard compound when analyzed by HPLC using a Rezex RSO-Oligosaccharide column. However, as shown below, the peak contained a mixture of FL isomers (Fig. 2). Maximum FL yields of 55% and 4% were obtained for Fuc2358 and Fuc5372, respectively (Fig. 1).

The transglycosylation reactions with the α-L-fucosidases Fuc2358 and Fuc5372 were concentrated and independently loaded on an ion-exclusion chromatography column. The purified products from the Fuc2358 and Fuc5372 reactions were named as F1 and F2, respectively (Fig. 2a and b), and analyzed by HPAEC-PAD using a Dionex system (Fig. 2c). The purified fraction F1 appeared as two peaks (Fig. 2c). The first peak exhibited the same retention time (17.8 min) as the 2′FL standard compound and the second peak showed a retention time (19.5 min) different from all available FL standards: 2′FL, 3FL, 6′FL (Fucα1-6Galβ1-4Glc), and DFL (Fucα1-2Galβ1-4(Fucα1-3)Glc). Similarly, fraction F2 from the Fuc5372 transfucosylation reaction was separated into the same two peaks as fraction F1 and two additional peaks with retention times of 11.0 min and 12.4 min, respectively (Fig. 2c). Interestingly, Fuc2358 produced larger amounts of 2′FL than Fuc5372 and produced more 2′FL than of the 19.5-min retention time product. Conversely, Fuc5372 produced more of the 19.5-min retention time product than of 2′FL.

**Fig. 3:**
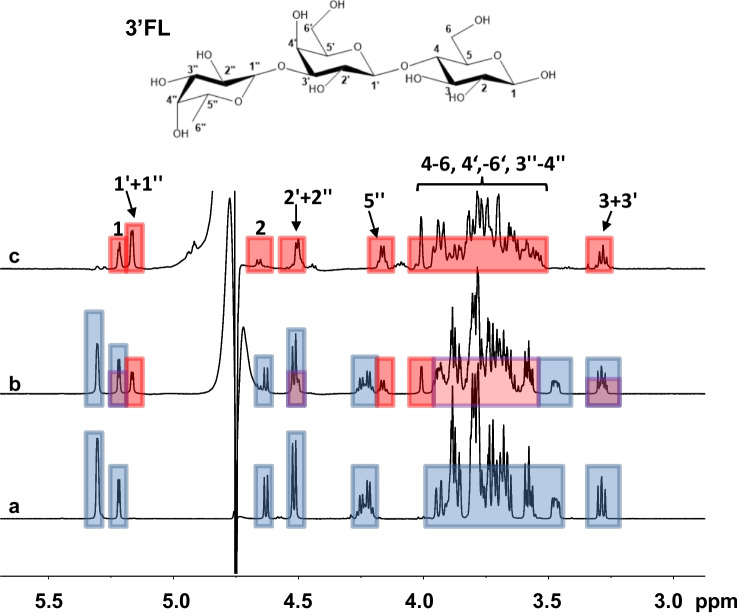
^1^H 1D NMR spectra of commercial 2′-fucosyllactose (2′FL) (**a**) and fractions F1 (**b**) and F2 (**c**) from Fuc2358 and Fuc5372, respectively. Spectra were acquired at 600 MHz. ns = 256. *T* = 25 °C in D_2_O. Signals corresponding to 2′FL compound are marked in blue. Common signals between F1 and F2 are marked in red and could be assigned to 3′-fucosyllactose (3′FL)

The purified F1 and F2 fractions were subjected to NMR analysis to further characterize them (Fig. 3). The structural analysis of F1 confirmed that Fuc2358 synthetized 2′FL. In addition, NMR spectra of F1 showed signal pattern shifts that correspond to 3’FL (Fucα1-3Galβ1-4Glc), which are also observed in F2 NMR spectra (Fig. 3).

**Fig. 4:**
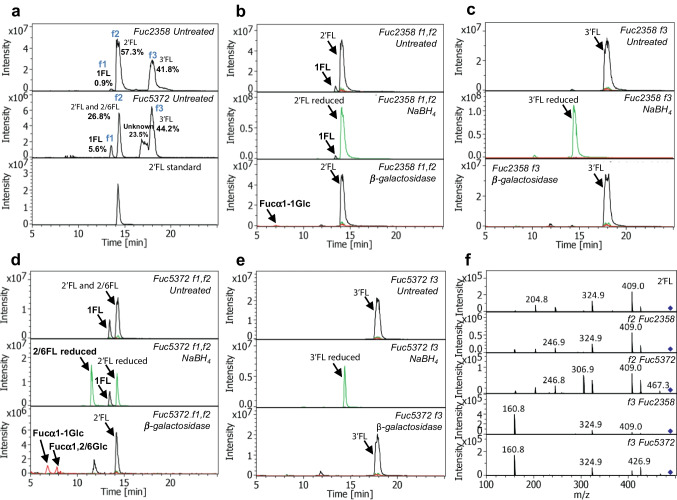
**a** LC-ESI-MS chromatograms of transfucosylation reactions with Fuc2358 and Fuc5372, and 2′FL standard. **b** Fuc2358 purified fractions f1 and f2. **c** Fuc2358 purified fraction f3. **d** Fuc5372 purified fractions f1 and f2. **e** Fuc5372 purified fraction f3. **f** MS^2^ fragmentation patterns of m/z 487.0 Da of the 2′FL standard and f2 and f3 peaks of Fuc2358 and Fuc5372. The parent ion [M-H]− is indicated by a diamond. For each fraction, the untreated fraction (top), the fraction treated with NaBH_4_ (middle), and the fraction incubated with the β-galactosidase from *Aspergillus oryzae* (bottom) are represented. Isomers present in the samples without treatment or not affected by the treatment are represented in black, reduced isomers are represented in green, and isomers hydrolyzed by the β-galactosidase are represented in red. The relative percentage of the fucosyllactose isomers produced is represented (**a**). All LC-ESI-MS chromatograms are combined extraction ion chromatograms (EIC) of deprotonated [M-H]^−^ and formate adducts [M-H + FA]^−^. 2′FL, 2′-fucosyllactose; 3′FL, 3′-fucosyllactose; 1FL, 1-fucosyllactose; 2/6FL, 2/6-fucosyllactose

For additional structural information on the FL isomers synthesized by Fuc2358 and Fuc5372, 4 h transfucosylation reactions were analyzed by LC-ESI-MS (Fig. 4a). Three common FL product peaks were observed for both α-L-fucosidases, which were purified and subjected to reduction reactions with NaBH_4_ or hydrolysis reactions with an *A. oryzae* β-galactosidase and further analyzed by LC-ESI-MS. The FL isomer contained in the first peak of Fuc2358 transfucosylation reaction (f1) was hydrolyzed by the β-galactosidase but not reduced, suggesting that it is 1-fucosyllactose (1FL; Galβ1-4Glc(Fucα1-1)) (Fig. 4b). The product obtained in the second peak of Fuc2358 (f2) underwent reduction but not hydrolysis (Fig. 4b), and showed the same retention time and fragmentation pattern as 2′FL standard (Fig. 4f). The third peak (f3) from the Fuc2358 transfucosylation reaction (Fig. 4c) remained unhydrolyzed by the β-galactosidase but was reduced upon treatment with NaBH_4_, suggesting that it is 3′FL, which was also observed in the F1 product of Fuc2358 by NMR. Regarding the Fuc5372 transfucosylation reaction, the analysis of the FL isomer present in the first peak (f1) showed that it is 1FL (Fig. 4d). The second peak of Fuc5372 (f2) resulted in a mixture of 2′FL and another product that was both reduced and hydrolyzed, indicating that fucose is located on the glucose moiety of lactose, but not at O1. Since previous results indicated that 3FL is not synthesized by Fuc5372, it could be either 2-fucosyllactose (2FL; Galβ1-4(Fucα1-2)Glc) or 6-fucosyllactose (6FL; Galβ1-4(Fucα1-6)Glc) (Fig. 4d). The fourth peak (f3) (Fig. 4e) was reduced but not hydrolyzed and showed the same retention time and fragmentation patterns as 3′FL in Fuc2358 transfucosylation reaction (Fig. 4f). The relative proportion of the FL isomers synthesized by the α-L-fucosidase Fuc2358 is 57.3% of 2′FL, 41.8% of 3′FL, and less than 1% of 1FL. Fuc5372 produces 44.2% of 3′FL, 26.8% of 2′FL, and2/6FL, 23.5% of unidentified FL, and 5.6% of 1FL (Fig. 4a).

### Structural modelling and rational design of mutants

**Fig. 5:**
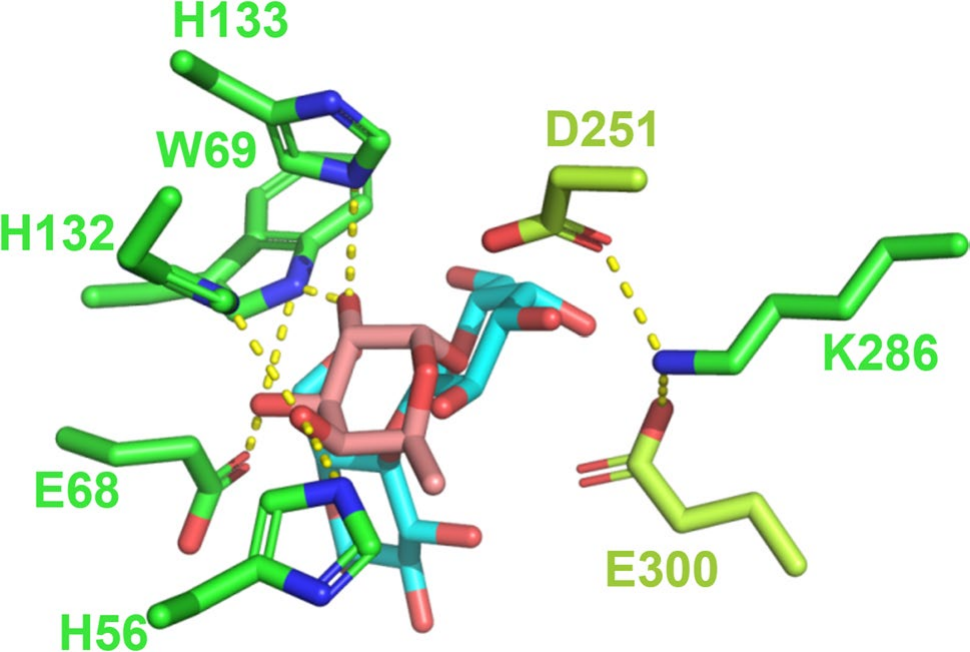
Catalytic pocket of Fuc2358 recombinant protein with the lactose (blue) docked and the α-L-fucose (pink) extracted from the crystal structure of the α-L-fucosidase E1_10125 from *Ruminococcus gnavus* mutant (PDB 6TR4) and superimposed. Only catalytic residues D251 and E300 (lime green), and putative substrate/product interacting residues (H56, E68, W69, H133, H132, and K286) are enhanced (green). Hydrogen bonds are shown as yellow dashed lines

The structural model of Fuc2358 with α-L-fucose and lactose (Fig. 5) confirms that Fuc2358 is a *syn*-protonating GH (Nerinckx et al. 2005). Although a successful docking for lactose and α-L-fucose was not obtained for Fuc5372, superimposed lactose and α-L-fucose indicated that this α-L-fucosidase is also a *syn* protonating GH (data not shown). Moreover, α-L-fucose is enveloped by the catalytic pocket of Fuc2358 and it can be observed that His56, His132, His133, and Trp69 provide hydrogen-bonded interactions with α-L-fucose, as also observed in the crystal structures of the α-L-fucosidases TmαFuc from *Thermotoga maritima* (Sulzenbacher et al. 2004), AlfC from *Lacticaseibacillus paracasei* (Klontz et al. 2020), and E1_10125 from *Ruminococcus gnavus* (Wu et al. 2021). Glu68 from Fuc2358 is also H-bonded to the α-L-fucose, as observed in TmαFuc (Sulzenbacher et al. 2004). Distinctly, Fuc2358 presents a Lys286 residue that may interact with the catalytic nucleophile and acid-base, whereas TmαFuc has an Arg residue interacting with the nucleophile and the acid-base in this position (Sulzenbacher et al. 2004). AlfC has an Arg in a similar position, but it does not interact with the nucleophile, and E1_10125 does not have any positively charged residue in this area.

**Fig. 6:**
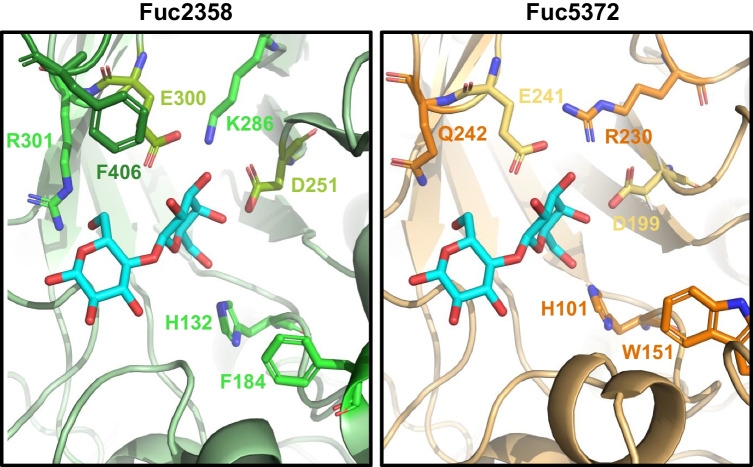
Active site structural alignments of the recombinant proteins Fuc2358 (green, left) and Fuc5372 (orange, right) structures obtained by AlphaFold2. Catalytic residues are indicated in lime green in Fuc2358 (E300 and D251) and yellow in Fuc5372 (E241 and D199). F406 from Fuc2358 is represented in forest green in Fuc2358. The lactose ligand is docked into Fuc2358 (cyan, left) and superimposed in Fuc5372 (cyan, right). Amino acid residues subjected to point mutation are indicated in green for Fuc2358 and orange for Fuc5372

Successful mutations in the α-L-fucosidase AlfB from *L. paracasei* (H80F and W130H) that increase its transfucosylation efficiency have been previously characterized (Teze et al. 2021). By structural and sequence alignment (Figs. S1 and S2), it was identified that the residue H80 in AlfB is homologous to H132 in Fuc2358 and H101 in Fuc5372. In the same way, residue W130 in AlfB is homologous to F184 in Fuc2358 and W151 in Fuc5372. Based on the structural alignment of Fuc2358 and Fuc5372 (Fig. 6), it was determined that Fuc2358 showed F406 very close to the acid-base residue (E300) and this residue is on a loop that is not present in Fuc5372. Two other residues closer than 4 Å to the lactose docked in Fuc2358 also differed between the two enzymes: R301 of Fuc2358 aligns with Q242 of Fuc5372, while the acid-base and nucleophile-interacting K286 of Fuc2358 aligns with R230 of Fuc5372.

Based on previous successful mutations in homologous residues of AlfB (Teze et al. 2021) and on the structural alignment differences between the Fuc2358 and Fuc5372 enzymes just described, 10 mutations were selected and tested for their hydrolytic and transfucosylation activity: Fuc2358-H132F, Fuc2358-F184H, Fuc2358-F184W, Fuc2358-R301Q, Fuc2358-K286R, Fuc5372-H101F, Fuc5372-W151H, Fuc5372-W151F, Fuc5372-Q242R, and Fuc5372-R230K.

### Effect of site-directed mutations on transfucosylation activity

The ratio of transglycosylation to hydrolysis should be maximized for efficient transglycosylation activity using glycosidases. Therefore, the hydrolytic activity of the constructed mutants was determined (Table 1). The results showed that the activity against pNP-Fuc decreased in all Fuc2358 mutants compared to Fuc2358-WT (Table 1). The hydrolytic activity also decreased in the Fuc5372-H101F, Fuc5372-W151H, and Fuc5372-R230K mutants, but increased in Fuc5372-W151F and Fuc5372-Q242R compared to Fuc5372-WT.

**Table 1:** Hydrolytic activity of Fuc2358 and Fuc5372 mutants

α-L-fucosidase	Relative hydrolytic activity on pNP-Fuc (%)^a^
Fuc2358-WT	100.0^a^
Fuc2358-H132F	0.7 ± 0.2^c^
Fuc2358-F184H	2.6 ± 0.4^c^
Fuc2358-F184W	25.6 ± 2.4^b^
Fuc2358-R301Q	5.4 ± 1.6^c^
Fuc2358-K286R	14.4 ± 11.6^b,c^
Fuc5372-WT	100.0^b^
Fuc5372-H101F	8.5 ± 0.2^c^
Fuc5372-W151H	42.6 ± 3.0^c^
Fuc5372-W151F	122.6 ± 2.4^b^
Fuc5372-Q242R	455.9 ± 44.2^a^
Fuc5372-R230K	2.2 ± 1.7^c^

^a^Hydrolytic activity is represented as a percentage of *p*-nitrophenyl-α-L-fucopyranoside (pNP-Fuc) hydrolyzed relative to their respective WT. Data presented are mean ± standard deviation values based on at least two replicates. Superscript letters (a–e) indicate significant differences (*p* < 0.05) for each parameter between variants of the same enzyme

**Fig. 7:**
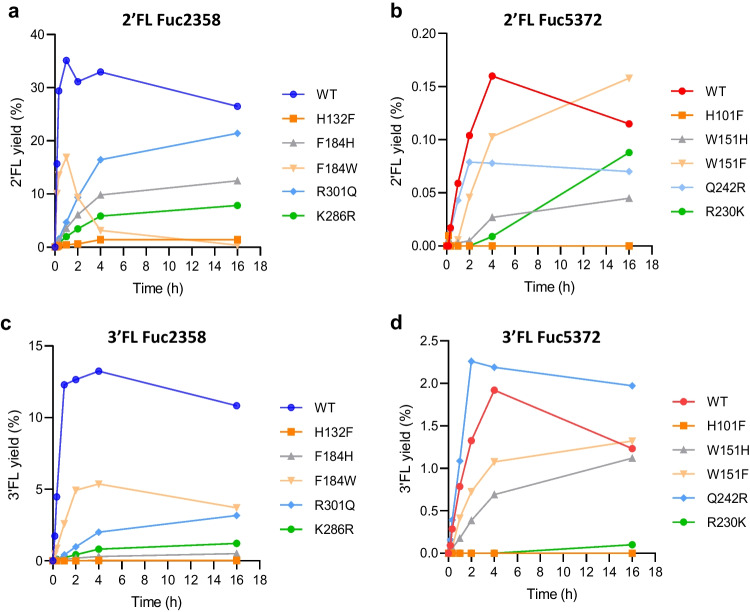
Time-dependent synthesis of 2′-fucosyllactose (2′FL; **a**, **b**) and 3′-fucosyllactose (3′FL; **c**, **d**) by transfucosylation reaction with 50 μg ml^−1^ of Fuc2358-WT (blue) and its respective mutants (**a**, **c**) and Fuc5372-WT (red) and its respective mutants (**b**, **d**). Mutations in homologous residues of both enzymes are represented in the same color: H132F and H101F in orange, F184H and W151H in gray, F184W and W151F in yellow, R301Q and Q242R in light blue, and K286R and R230K in green. Product yields are expressed as a percentage of the donor concentration (*p*-nitrophenyl-α-L-fucopyranoside 50 mM) and 3′FL product is calculated as equivalents of 2′FL

Regarding transfucosylation activity, the wild-type enzymes Fuc2358 and Fuc5372 reached maximum 2′FL yields of 35.13% at 2 h of reaction and 0.16% at 4 h of reaction, respectively (Fig. 7a and b). Maximum 3′FL yields of 13.24% and 1.92% were also obtained at 4 h of reaction for Fuc2358 and Fuc5372, respectively (Fig. 7c and d). All the Fuc2358 mutants analyzed showed lower 2′FL yields than the wild-type Fuc2358 (Fig. 7a and Table 2). Nevertheless, Fuc2358-F184H and Fuc5372-R230K showed significantly higher transglycosylation/hydrolysis ratio for the synthesis of 2′FL than their respective WT at 4 h of reaction (Table 2). Notably, Fuc2358-F184W exhibited a significant decrease in 2′FL yields after 1 h of reaction (Fig. 7a) and its transglycosylation/hydrolysis ratio for 2’FL synthesis at 4 h was significantly lower than the Fuc2358-WT transglycosylation/hydrolysis ratio (Table 2). Interestingly, the percentage of 3′FL yields did not decrease proportionally in this mutant, which is reflected in a lower 2'FL/3′FL ratio. Except for Fuc2358-F184W, all Fuc2358 mutants showed a higher 2′FL/3′FL ratio than Fuc2358-WT (Table 2).

**Table 2:** Transfucosylation yields, transglycosylation/hydrolysis ratios, and regioselectivity ratio of Fuc2358-WT, Fuc5372-WT, and their respective mutants at 4 h of reaction

α-L-fucosidase	2′FL yield (%)^a^	Relative 2′FL yield (%)^b^	2′FL yield/pNP-Fuc_hydrolyzed_ ratio^c^	3′FL yield (%)^a^	Relative 3′FL yield (%)^b,d^	3′FL yield/pNP-Fuc_hydrolyzed_ ratio^c^	2′FL/3′FL ratio^e^
Fuc2358-WT	32.99 ± 0.02^a^	100.0 ± 0.1^a^	0.377 ± 0.093^d^	9.03 ± 2.31^a^	100.0 ± 25.6^a^	0.097 ± 0.002^a^	3.91 ± 1.00^c^
Fuc2358-H132F	0.69 ± 0.04^c^	2.1 ± 0.1^c^	0.581 ± 0.031^bc^	0.03 ± 0.01^c^	0.3 ± 0.1^c^	0.020 ± 0.005^d^	30.64 ± 11.92^ab^
Fuc2358-F184H	10.99 ± 1.16^bc^	33.3 ± 3.5^bc^	0.780 ± 0.006^ab^	0.45 ± 0.14^b,c^	4.9 ± 1.5^bc^	0.031 ± 0.005^d^	26.31 ± 5.38^abc^
Fuc2358-F184W	8.26 ± 5.10^bc^	25.0 ± 15.5^bc^	0.073 ± 0.036^e^	7.25 ± 1.88^a^	80.3 ± 20.8^a^	0.069 ± 0.005^c^	1.03 ± 0.44^c^
Fuc2358-R301Q	15.92 ± 0.52^bc^	48.3 ± 1.6^bc^	0.662 ± 0.014^abc^	1.98 ± 0.03^bc^	21.9 ± 0.3^bc^	0.083 ± 0.003^b^	8.05 ± 0.14^bc^
Fuc2358-K286R	8.80 ± 2.9^bc^	26.7 ± 9.0^bc^	0.764 ± 0.005^ab^	1.22 ± 0.40^bc^	13.5 ± 4.5^bc^	0.106 ± 0.001^a^	7.20 ± 0.04^c^
Fuc5372-WT	0.115 ± 0.032^ab^	100.0 ± 22.1^ab^	0.0037 ± 0.0003^b^	1.440 ± 0.393^b^	100.0 ± 27.3^b^	0.043 ± 0.004^b^	0.081 ± 0.008^b^
Fuc5372-H101F	0.0	0.0	-	0.0	0.0	-	-
Fuc5372-W151H	0.024 ± 0.001^bc^	20.8 ± 0.8^bc^	0.0036 ± 0 .0005^b^	0.589 ± 0.050^d^	40.9 ± 3.5^d^	0.079 ± 0.012^a^	0.041 ± 0.005^c^
Fuc5372-W151F	0.137 ± 0.043^ab^	119.1 ± 37.0^ab^	0.0048 ± 0.0013^b^	1.134 ± 0.190^c^	78.7 ± 13.2^c^	0.039 ± 0.004^b^	0.119± 0.020^a^
Fuc5372-Q242R	0.095 ± 0.014^abc^	82.4 ± 12.4^abc^	0.0015 ± 0.0004^b^	2.354 ± 0.071^a^	163.4 ± 4.9^a^	0.034 ± 0.005^b^	0.040 ± 0.005^c^
Fuc5372-R230K	0.007 ± 0.002^bc^	6.4 ± 1.6^bc^	0.0213 ± 0.0093^a^	0.0	0.0	-	-

^a^Yields are expressed as a percentage of donor concentration (*p*-nitrophenyl-α-L-fucopyranoside (pNP-Fuc) 50 mM)

^b^Yields are expressed as a percentage of donor concentration relative to their respective WT

^c^Tranglycosylation/hydrolysis ratios are expressed as 2′-fucosyllactose (2′FL) or 3′-fucosyllactose (3′FL) yields to pNP-Fuc hydrolyzed

^d^3′FL concentration is calculated as equivalents of 2′FL, using the 2′FL standard

^e^Regioselectivity ratio is expressed as 2′FL yields to 3′FL yields

Data presented are mean ± standard deviation values based on at least two replicates. Superscript letters (a–e) indicate significant differences (*p* < 0.05) for each parameter (column-wise) between variants of the same enzyme

The Fuc5372-Q242R mutant showed increased 3′FL yields compared to Fuc5372-WT (Fig. 7d and Table 2). Fuc5372-R230K mutant showed a higher transglycosylation/hydrolysis ratio for the synthesis of 2′FL and Fuc5372-W151H for the synthesis of 3’FL respect to the wild-type (Table 2). Regarding the regioselectivity ratio, the Fuc5372-W151H and Fuc5372-W151F mutants showed a significant lower and higher 2′FL/3′FL ratio, respectively, compared with Fuc5372-WT (Table 2).

Transfucosylation reactions using a 10-fold higher enzyme dosage (500 μg ml^−1^) were performed with the enzymes Fuc2358-R301Q, Fuc2358-K286R, Fuc5372-W151H, and Fuc5372-W151F, which are among the mutants with higher transglycosylation/hydrolysis ratios for the synthesis of both 2′FL and 3′FL. The results showed that Fuc2358-K286R and Fuc5372-W151F synthesized higher yields of 2′FL than Fuc2358-WT and Fuc5372-WT, respectively (Table 3). Moreover, all the mutants tested produced higher 3′FL yields than their respective WT. In all the cases, the increase in the product yields is correlated with an increase in the transglycosylation/hydrolysis ratio compared to their respective WT (Table 3).

**Table 3:** Transfucosylation yields and transglycosylation/hydrolysis ratios of Fuc2358 and Fuc5372 mutants at 4 h of reaction using 500 μg ml^−1^ of enzyme

α-L-fucosidase	2′FL yield (%)^a^	2′FL yield/pNP-Fuc_hydrolyzed_ ratio^b^	3′FL yield (%)^a,c^	3′FL yield/pNP-Fuc_hydrolyzed_ ratio^b^
Fuc2358-WT^d^	32.99	0.377	9.03	0.097
Fuc2358-R301Q	13.92	0.205	14.62	0.215
Fuc2358-K286R	42.95	0.597	14.15	0.197
Fuc5372-WT^d^	0.12	0.004	1.44	0.043
Fuc5372-W151H	0.08	0.002	5.40	0.127
Fuc5372-W151F	0.24	0.007	3.02	0.080

^a^Yields are expressed as a percentage of donor concentration (*p*-nitrophenyl-α-L-fucopyranoside (pNP-Fuc) 50 mM)

^b^Tranglycosylation/hydrolysis ratios are expressed as 2′-fucosyllactose (2′FL) or 3′-fucosyllactose (3´FL) yields to pNP-Fuc hydrolyzed

^c^3′FL concentration is calculated as equivalents of 2′FL, using the 2′FL standard

^d^The values for Fuc2358-WT and Fuc5372-WT correspond to 50 μg ml^−1^ of enzyme, which is the protein concentration tested at which the maximum yield is obtained

## Discussion

The gut bacteria possess the ability to digest the fucose moiety of fucosylated glycans by expressing a wide array of α-L-fucosidases with different linkage specificities (Sakanaka et al. 2019; Zuñiga et al. 2018; Zuñiga et al. 2020). This study focuses on the utilization of GH29 α-L-fucosidases, previously isolated from the infant gut microbial metagenome of breastfed infants (Moya-Gonzalvez et al. 2022), to produce fucosylated glycans through transfucosylation reactions, and the application of protein engineering based on rational design to improve their transfucosylation activity. The α-L-fucosidases Fuc2358 and Fuc5372 were able to synthesize different FL isomers, including the principal fucosylated HMO 2′FL, in reactions using pNP-Fuc as donor substrate and lactose as acceptor substrate. Both α-L-fucosidases belong to the GH29 subfamily A, which presents a rather relaxed regioselectivity for the donor (Zeuner et al. 2018a). Maximum 2′FL yields of 35% were obtained for Fuc2358. This trisaccharide has also been synthesized enzymatically using xyloglucan as donor and lactose as acceptor, obtaining maximum transfucosylation yields of 16% (Zeuner et al. 2020). α-L-fucosidases GH29A isolated from a soil metagenome have also been used in transfucosylation reactions using pNP-Fuc as donor and lactose as acceptor substrate (Lezyk et al. 2016) with very low 2′FL yields ranging from 0.15 to 0.35%. In the same work, maximum 2′FL yields of 0.95% were obtained with the α-L-fucosidase TmαFuc from *T. maritima*. 2′FL is one of the major neutral HMOs detected in the milk of secretor mothers (Thurl et al. 2017) and several positive effects have been reported when adding this oligosaccharide to infant formulas (Reverri et al. 2018). Thus, the results obtained here regarding the transfucosylation activity of Fuc2358, which showed high 2′FL yields compared to previously described enzymes, are of great interest, not least for future expansion to more complex α-1,2-fucosides such as LNFP I and LDNFH I.

In addition to 2′FL, both α-L-fucosidases, Fuc2358 and Fuc5372, synthesize other FL isomers including the non-HMO 3′FL and the non-reducing 1FL. Fuc2358 synthesizes higher amounts of 2′FL than 3′FL, while Fuc5372 is just the opposite. Moreover, considering all the fucosylated glycans produced by both enzymes, Fuc5372 synthesizes lower amounts of FUS than Fuc2358. Different levels of regioselectivity have been already observed among the GH29A family and non-HMO oligosaccharides (α-1,1-, α-1,3-, α-1,4-, or α-1,6-fucosylated) have been synthesized when trying to obtain α-1,2-fucosylated products (Perna et al. 2023; Thogersen et al. 2020; Usvalampi et al. 2020; Zeuner and Meyer 2020; Zeuner et al. 2018a). Although studies to evaluate their potential beneficial effects should be performed, these non-HMOs could have a biotechnological application as antiadhesive antimicrobials, as previously observed for other human milk glycans (Newburg et al. 2005; Ray et al. 2019), but with the putative advantage of not being metabolized by gut bacteria.

The structural models of Fuc2358 and Fuc5372 confirmed the *syn* protonating as described previously for other GH29 α-L-fucosidases (Sakurama et al. 2012a), and conserved residues that interact with α-L-fucose in other enzymes (Klontz et al. 2020; Sulzenbacher et al. 2004; Wu et al. 2021) are also present in both α-L-fucosidases. Transglycosylation reactions take place in competition with the hydrolytic reaction, resulting in low product yields. Therefore, strategies have been developed to force the balance between the transglycosylation and hydrolysis rates to overcome this limitation. Protein engineering has been widely used with GHs to increase transglycosylation efficiency (Jamek et al. 2018; Nyffenegger et al. 2017; Teze et al. 2014). This has been improved in several GHs by targeting a small number of conserved active-site residues using sequence conservation analysis (Teze et al. 2021), and these mutations can be transposed to GHs belonging to the same GH family (Teze et al. 2015; Teze et al. 2014; Yang et al. 2017). In particular, protein engineering has been already used to improve the transfucosylation efficiency in GH29A α-L-fucosidases (Teze et al. 2021; Zeuner et al. 2020). Fuc2358-H132F and Fuc2358-F184H mutants were constructed here based on previous successful mutations in AlfB from *L. paracasei* (Teze et al. 2021). The mutated residues increased the transglycosylation/hydrolysis ratio for the synthesis of 2′FL in Fuc2358 mutants compared to Fuc2358-WT. A lower hydrolytic activity of these mutants against pNP-Fuc compared to the Fuc2358-WT could explain the increase in the ratio. This is in agreement with previous results (Teze et al. 2021), where the increase in the transglycosylation/hydrolysis ratio of the mutants was not correlated with a lower secondary hydrolysis but with a higher transfucosylation rate or a lower pNP-Fuc hydrolysis rate. However, lower transglycosylation/hydrolysis ratios for the synthesis of 3′FL were obtained for both mutants compared to Fuc2358-WT. Interestingly, opposite to Fuc2358-F184H, Fuc5372-W151H, which has the mutation in the homolog residue, showed a higher transglycosylation/hydrolysis ratio for the synthesis of 3′FL than Fuc5372-WT at 4 h. These results indicate that this residue has an effect on the regioselectivity of both enzymes, and histidine has the opposite effect on each enzyme.

Although there are certain active site residues critical for the enzymatic activity that are conserved (Wu et al. 2023; You et al. 2019), there can be variations in the amino acid sequence and the structure between α-L-fucosidases belonging to the same GH family. These variations might contribute to the substrate binding and the stabilization of the transition states (Kovalova et al. 2019), leading to differences in transglycosylation activity between enzymes. Indeed, Fuc5372-W151F showed higher yields of both 2′FL and 3′FL than Fuc5372-WT when the reaction was performed with 500 μg mL^−1^. Notably, Fuc2358 has a phenylalanine residue (F184) in this otherwise conserved position; Phe is among the replacements suggested for Trp (the other is His) when following the strategy of replacing conserved residues with structurally homologous residues (Teze et al. 2021; Vuillemin et al. 2021). Indeed, mutation F184W in Fuc2358, whose residue is homolog to W151 in Fuc5372, had the opposite effect, exhibiting a significant decrease in 2′FL yields. It has been previously described that aromatic residues surrounding the active site of different GH29 α-L-fucosidases contribute to substrate binding in the active site, promoting secondary hydrolysis (Jimenez-Perez et al. 2023; Kovalova et al. 2019). The side chain of tryptophan is larger and more complex than that of phenylalanine, which can result in an increased steric hindrance, causing higher retention of the product in the active site and thus higher degree of product hydrolysis. This effect could explain the increase in the synthesis yields of 2′FL and 3′FL in Fuc5372-W151F compared to Fuc5372-WT, and the decrease in 2′FL and 3′FL yields in Fuc2358-F184W mutant compared to Fuc2358-WT. Moreover, the 2′FL/3′FL ratio increases in Fuc5372-W151F and decreases in Fuc2358-F184W compared to their respective WT. These results suggested that this residue is involved in the regioselectivity of both enzymes as mentioned above. Interestingly, they also showed that phenylalanine increases the selectivity for α-1,2 linkages and tryptophan for α-1,3 linkages.

Based on sequence and structural alignments, Fuc2358 was shown to have a Lys286 residue that differs from the Arg residue found in other described α-L-fucosidases (Sulzenbacher et al. 2004), including Fuc5372 (R230). Due to its direct interaction with the acid-base and the nucleophile, the effect of this residue on the hydrolytic and transfucosylation activity of both enzymes was studied. Fuc5372-R230K reached almost the same amount of 2′FL as Fuc5372-WT when incubating overnight. Arginine is a basic residue that is also described to strengthen the interaction between the enzyme and the substrate (Jimenez-Perez et al. 2023; Kovalova et al. 2019). Lysine could reduce the interaction, producing a decrease in hydrolysis, which would explain the higher transglycosylation/hydrolysis ratio obtained for the synthesis of 2′FL in Fuc5372-R230K compared to Fuc5372-WT. However, the same effect is observed when the opposite mutation is introduced in Fuc2358-K268R. These results suggested that the effect on the ratio could be caused by the subtle change in amino acid structure, rather than the nature of the amino acid residue itself. Indeed, the subtle changes obtained by exchanging arginine with lysine are among those suggested to destabilize the transition state when conservatively replacing conserved active site residues, which is commonly more harmful to hydrolysis than to transglycosylation (Teze et al. 2021).

Protein folding is better conserved than amino acid sequence between GHs belonging to the same family. In this study, AlphaFold2 structures of Fuc2358 and Fuc5372 showed that Fuc2358 exhibits a loop that is not present in Fuc5372. This loop has a phenylalanine at position 406, which is closer than 4 Å to the lactose docked into Fuc2358 and could be interacting with the acid-base residue (E300) and with R301 (Fig. 6). Arginine is a polar and positively charged residue that could bring protons to the acid-base residue, promoting the hydrolysis of the substrate (Zeuner and Meyer 2020). F406 seems to be blocking the interaction between the R301 and the acid-base residue. This loop has not been previously reported in α-L-fucosidases from the GH29A subfamily. Fuc5372 has a glutamine residue (Q242) that is structurally homologous to R301 in Fuc2358, and there is no residue present between the Q242 and the acid-base (E241) that could be blocking their interaction, although glutamine cannot deliver protons to the acid-base residue in the same way as arginine. Indeed, Fuc5372-Q242R increased the hydrolytic activity against pNP-Fuc by 455.9 ± 44.2% compared to Fuc5372-WT. Moreover, the transfucosylation/hydrolysis ratios for the synthesis of 2’FL and 3′FL were lower compared to Fuc5372-WT, which can be explained by higher hydrolysis of the pNP-Fuc. Even so, this mutant produced higher yields of 3′FL than Fuc5372-WT. Conversely, the transfucosylation/hydrolysis ratio for the synthesis of 2′FL showed a tendency to increase in the Fuc2358-R301Q mutant, possibly due to a lower hydrolytic activity since glutamine does not deliver protons to the acid-base residue.

The results obtained in the present work show the different linkage specificity in the transfucosylation activity of α-L-fucosidases isolated from the intestinal microbiota of breastfed infants. Bacteria in this environment have emerged as a valuable reservoir of α-L-fucosidases with possible application in the production of FUS through transfucosylation reactions. The results also highlight the potential of protein engineering based on structural modeling for transfucosylation efficiency improvement and regioselectivity modulation.

## Supplementary information

ESM 1 (PDF 219 kb)

## Data Availability

All data generated or analyzed during this study are included in this published article and its supplementary information files.
