# Supplementary material for: Synthesis of fucosyllactose using α-L-fucosidases GH29 from infant gut microbial metagenome
Source: Appl Microbiol Biotechnol. 2024 May 21;108(1):338. doi: 10.1007/s00253-024-13178-3 (PMC11108932; doi:10.1007/s00253-024-13178-3)
Supplement: Supplementary file 1 — (PDF 219 kb) [file 253_2024_13178_MOESM1_ESM.pdf]

## Supplementary Information

# Synthesis of fucosyllactose using α-L-fucosidases GH29 from infant gut microbial metagenome

**Eva M. Moya-Gonzálvez[1], Birgitte Zeuner[2], Albert Th. Thorhallsson[2], Jesper Holck[2], Martina Palomino-Schätzlein[3], Jesús Rodríguez-Díaz[4], Anne S. Meyer[2] and María J. Yebra[1*]**

[1]Laboratorio de Bacterias Lácticas y Probióticos, Departamento de Biotecnología de Alimentos, Instituto de Agroquímica y Tecnología de Alimentos (IATA-CSIC), Valencia, Spain.
[2]Protein Chemistry and Enzyme Technology, Department of Biotechnology and Biomedicine, Technical University of Denmark, Lyngby, Denmark.
[3]ProtoQSAR, CEEI, Parque Tecnológico Valencia, Paterna 46980, Spain.
[4]Departamento de Microbiología, Facultad de Medicina, Universidad de Valencia, Valencia, Spain.

*Address correspondence to María J. Yebra, yebra@iata.csic.es

Applied Microbiology and Biotechnology

**Table S1**. Primers used in this study.

| Primer name | Sequence (5'→3')[a] |
|---|---|
| **Fuc5372-WT forward** | 5'-CTGGGATCCCAAGATACCCTGCAAAATGAGACC |
| **Fuc5372-WT reverse** | 5'-ATTAAGCTTGGCTGCAGGTCGACCCTAGCTAGG |
| **Fuc5372-H101F forward** | 5'CCACCAAA**TTC**CACGATGGTG |
| **Fuc5372-H101F reverse** | 5'CACCATCGTG**GAA**TTTGGTGG |
| **Fuc5372-W151F forward** | 5'CCCTGCTGGAC**TTT**TCACACCCTGAC |
| **Fuc5372-W151F reverse** | 5'GGTGGCT**AAA**GTCCAGCAGGC |
| **Fuc5372-W151H forward** | 5'CCCTGCTGGAC**CAT**TCACACCCTGAC |
| **Fuc5372-W151H reverse** | 5'GGTGGCT**ATG**GTCCAGCAGG |
| **Fuc5372-R230K forward** | 5'CATCAACTCC**AAA**ATCCAAGGCTACGGCG |
| **Fuc5372-R230K reverse** | 5'CGCCGTAGCCTTGGAT**TTT**GGAGTTGATG |
| **Fuc5372-Q242R forward** | 5'CTACTCCTGAG**CGC**GGTGTCCCTG |
| **Fuc5372-Q242R reverse** | 5'CAGGGACACC**GCG**CTCAGGAGTAG |
| **Fuc2358-WT forward** | 5'-TTTTGCATGCATGATAAGCCTTGAAGAGATTG |
| **Fuc2358-WT reverse** | 5'-TTTTAAGCTTCTAAAAGCTGATGCGCAAAAC |
| **Fuc2358-H132F forward** | 5'GTTGCGGAG**TTC**CACGATGG |
| **Fuc2358-H132F reverse** | 5'CCATCGTG**GAA**CTCCGCAAC |
| **Fuc2358-F184H forward** | 5'CACCAGTTT**CAC**TTTTCACATG |
| **Fuc2358-F184H reverse** | 5'CATGTGAAAA**GTG**AAACTGGTG |
| **Fuc2358-F184W forward** | 5'CACCAGTTT**TGG**TTTTCACATG |
| **Fuc2358-F184W reverse** | 5'CATGTGAAAA**CCA**AAACTGGTG |
| **Fuc2358-K286R forward** | 5'GTTTGTTAC**CGC**CAGGATGCC |
| **Fuc2358-K286E reverse** | 5'GGCATCCTG**GCG**GTAACAAAC |
| **Fuc2358-R301Q forward** | 5'AGATGGAG**CAG**GGAGGCTACG |
| **Fuc2358-R301Q reverse** | 5'CGTAGCCTCC**CTG**CTCCATCT |

[a]Letters in bold indicate nucleotides that have been changed in the sequences of the genes that encode the wild-type enzymes to construct the mutant enzymes.

**Table S2**. Strains and plasmids used in this study.

| Strain or plasmid | Relevant genotype or properties | Source |
|---|---|---|
| ***E.coli* strains** | | |
| **DH10B** | F-*endA1 recA1 galE15 galK16 nupG rpsL* | Invitrogen |
| **PE187** | DH10B containing pQE80fuc2358 | Moya-Gonzálvez et al. (2022) |
| **E164** | DH10B containing pQE80fuc2358-R301Q | This work |
| **E165** | DH10B containing pQE80fuc2358-F184H | This work |
| **E166** | DH10B containing pQE80fuc2358-F184W | This work |
| **E167** | DH10B containing pQE80fuc2358-K286R | This work |
| **E168** | DH10B containing pQE80fuc2358-H132F | This work |
| **PE188** | DH10B containing pQE80fuc5372 | Moya-Gonzálvez et al. (2022) |
| **E159** | DH10B containing pQE80fuc5372-H101F | This work |
| **E160** | DH10B containing pQE80fuc5372-Q242R | This work |
| **E161** | DH10B containing pQE80fuc5372-R230K | This work |
| **E162** | DH10B containing pQE80fuc5372-W151H | This work |
| **E163** | DH10B containing pQE80fuc5372-W151F | This work |
| | | |
| **Plasmids** | | |
| **pQE80** | *E. coli* expression vector; $Amp^R$ | Qiagen |
| **pQE80fuc2358** | pQE80 containing *fuc2358* coding region | Moya-Gonzálvez et al. (2022) |
| **pQE80fuc2358-R301Q** | pQE80 containing *fuc2358*-R301Q coding region | This work |
| **pQE80fuc2358-F184H** | pQE80 containing *fuc2358*-F184H coding region | This work |
| **pQE80fuc2358-F184W** | pQE80 containing *fuc2358*-F184W coding region | This work |
| **pQE80fuc2358-K286R** | pQE80 containing *fuc2358*-K286R coding region | This work |
| **pQE80fuc2358-H132F** | pQE80 containing *fuc2358*-H132F coding region | This work |
| **pQE80fuc5372** | pQE80 containing *fuc5372* coding region | Moya-Gonzálvez et al. (2022) |
| **pQE80fuc5372-H101F** | pQE80 containing *fuc5372*-H101F coding region | This work |
| **pQE80fuc5372-Q242R** | pQE80 containing *fuc5372*-Q242R coding region | This work |
| **pQE80fuc5372-R230K** | pQE80 containing *fuc5372*-R230K coding region | This work |
| **pQE80fuc5372-W151H** | pQE80 containing *fuc5372*-W151H coding region | This work |
| **pQE80fuc5372-W151F** | pQE80 containing *fuc5372*-W151F coding region | This work |

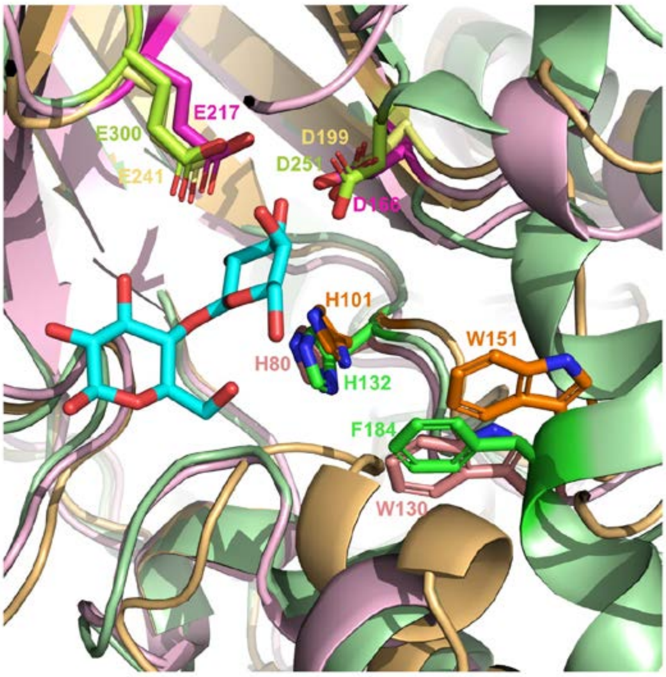


**Fig. S1**. Structural alignment of Fuc2358 (pale green), Fuc5372 (light orange) and AlfB from *Lacticaseibacillus paracasei* (ex-*Lactobacillus casei*) structures obtained by AlphaFold2, indicating the catalytic residues (lime green for Fuc2358, yellow for Fuc5372 and magenta for AlfB). Amino acid residues subjected to point mutation are indicated in green (Fuc2358) orange (Fuc5372) and violet (AlfB).

```
FgFCO1          PEAIIKIVKGSGARFAGPVAEHHDGFSMWDSK-VN---EWNPV-----NYGPKLDLVKLW   179
NixE            PTGWAKLFRESGARYVVPVAEHHDGFALYDSK-LS---DWTAM-----KMGPKRDLLGEL   218
Fuc2358         PASWLDLFQKAGAQYLFPVAEHHDGFQMYAST-LS---PYNSL-----EMGPRRDVLGEL   161
FoFCO1          AEDWMNLVADAGAQYVVPVTKHHDGWALFDFP-ESV-SKRSTV-----HYGPKRDFVKEL   293
DdFuc           ANEWASIIEKSGAKYVVLTSKHHEGYTLWNSE-QSW-N-WNSV-----ETGPGIDIVGEL   158
DmFuca          ATKWALLFKDSGARYVVLTSKHHDGFTLWPSK-NSY-G-WNSM-----DVGPKRDIVKEL   165
PapFuC          PKTFAEIIEASGAKYVVLTSKHHEGFTNWPSK-YSW-N-WNSM-----DVGPKRDLVGDL   163
RnFucA1         PEEWADLFQAAGAKYVVLTAKHHEGFTNWPSA-VSW-N-WNSK-----DVGPHRDLVGEL   164
ClFuc           PDTWADLFQAAGARYVVLTTKHHEGFTNWPSS-VSW-N-WNSN-----DVGPHRDLVGEL   168
FucA1           PEEWADLFQAAGAKYVVLTTKHHEGFTNWPSP-VSW-N-WNSK-----DVGPHRDLVGEL   163
AlfA            VQAWAAAFANAGAKYVVIVTKHHDGFVMFDPH-TKN-P-YE-P-----DYHLNFDFVGEL   157
TmaFuc          PQEWADLFKKAGAKYVIPTTKHHDGFCLWGTK-Y-T-D-FNSV-----KRGPKRDLVGDL   157
Blon0248        PADMADLFARAGARYFVPTTKHHEGITLWKAP-DND-G-WNTV-----DRGPHRDLVKEF   159
Blon0426        PADMADLFARAGARYFVPTTKHHEGITLWKAP-DND-G-WNTV-----DRGPHRDLVKEF   159
Fuc35B          TDQWIHTAKSLGAKYAVLVAKHCSGFSLWPTT-AHE---YSIK-NSPYKN-GKGDIVAEF   131
BbAfcB          TDQWAKSFADGGFKMIMVTVKHHDGFELYDSR-YNTEHDWANT-AVAKRT-GEKDLFRKI   612
BT2192          CRQWMQTLKAAGIPAAILTAKHADGFCLWPSK-YTD---YSVK-NAAWKN-GKGDVVREF   131
Fuc18           CEQWVQTFVNSGMKGVILTAKHHDGFCLWPTQ-LTE---YCIR-NTPYKD-GKGDIVREL   133
BiAfcB          VDQWMDALVAGGMAGVILTCKHHDGFCLWPSR-LTR---HTVA-SSPWRE-GKGDLVREV   117
BT1625          AEQWVRTLKDAGFKMVLLTAKHHDGFCLWPTA-TTK---HSVA-SSPWKN-GQGDVVKEL   149
Fuc39           AEQWVRTLKDAGFKMVLLTAKHHDGFCLWPTA-TTK---HSVA-SSPWKN-GQGDVVKEL   150
BT4136          AEQWVSNLKEAGFKMVILTAKHHDGFCLWPTA-TTK---HSVA-SSSWKN-GQGDVVKEL   149
AtFUC1          ASQWVQIAKDSGFSRVILTAKHHDGFCLWPSE-YTD---YSVK-SSQWRN-GAGDVVAEL   136
CpAfc2          ADQWVKTLADAGFGRVILTAKHHDGFCLWDSA-YTK---HDVA-SSPWKN-GKGDVVKEV   146
SpGH29          AREWVRVLKETGFKKLILVVKHHDGFVLYPTA-HTD---YSVK-VSPWRR-GKGDLLLEV   109
Fuc1584         ADAIVSLAKEAGMRSIIITTKHHDGFCMFRTA-TTD---YNSYDATPG----KRDFIKEM   139
AlfC            AKKIVALAKQCGMQYLVVTTKHHDGFAMYHSK-VDA---YNVYDATPF----HRDIIGEL   117
Fuc35A          AKKWVRAAKHAGMKYLVYTTKHHDGFAMYNSA-CSD---YNIVKCTPF----AKDPLKEL   137
Fuc30           CKQWAKAAKSANMTYGCLTTKHHSGFCIWDTK-TTD---YSVM-SSPF----KRDVVKEY   121
Fuc19A          PAQWAEVTKQAGMKYMIFTTKHHDGFCMFDSQ-YTD---FSIS-RCAFKEHPKADVAKYV   165
AlfB            AKKIAHAAKAVGAKYIVLTTKHHEGFFLYDTKGLSD---FDVM-HAPA----RRDLIAEF   110
BT2970          AKKWAKMAKEMGTKYVKITTKHHEGFCLWPSK-YTK---YTVA-NTPY----KRDILGEL   164
Fuc5372         PKAWVDLIKESGAKYTVITTKHHDGVALWDTK-VGD---ISVVKSTPA----KRDLIAPF   131
TfFuc1          AAEWVAAIKASGAKYITITSRHHDGFSMYATQ-QSD---YNIVDATPF----KRDVIHEL   138
Mfuc6           PKEWARLAKRVGMQYAIFTSKHHDGYAMYHTA-END---FSIG-HSPY----GKDIVRSF   115
Mfuc5           PAEWAKAARKAGMKYAVMTSKHHDGFCLFDSQ-YTD---YKAT-NTPA----GRDLVREY   107
aLfuk1          PKAWAKAAKAAGMKYAVMTTKHHDGFCLFDSA-LTD---YKAT-NTPA----GRDLIREY   111
Fuc193          PEEWAYMAKAAGVKYMVFTTKHHDGFCMWDSK-YTD---YKIT-NTPY----KKDILKPL   140
Mfuc2           PAAWARDARNAGMKYVVVTTKHHDGFCLWDSA-LTD---YKAP-NTPA----GRDLLRPL   113
Mfuc7           AGEWARQARDAGMKYVVMTTKHHEGFCMFDSR-YTD---YKCT-NTPA----GRDLVREY   114
Mfuc1           PRLWARAAREAGMKYVVLTTKHHEGFCLWDSQ-FTD---YKVT-NTPY----GKDLLTPF   113
Mfuc4           PRKWARAAREAGMKYVVLTTKHHEGFCLWDSA-YTD---YKAS-NTPY----GKDLLKPF   112
                           .      . .* .*   :                         *
```

**Fig. S2.** Continues on the next page.

```
FgFCO1        ADLVRE--NDMKLVIAMHQAYNYNGFFQWA----PKTNDTS-LQKLL------------  219
NixE          STAIRA--QGLHFGLSSHRAEHN-WFFDGGRKFDSDVNDPR-YAALYGPAQ---VRLPGK  271
Fuc2358       REEAEK--RGLHFCTSSHRAEHQ-FFFSHGKEFTSDISQEVPRNSLYWPAE---PEP--K  213
FoFCO1        LDVAKAKHPEIRRGTYFS-M--PEWFNPAY----AKYYWDQHYKEIYWGRP---PTNPY-  342
DdFuc         TKSVKN--MGLHMGLYHS-L--FEWFNPLY-------LADAET----GKNP---T-----  194
DmFuca        AAAIRKE-SDLRFGLYYS-L--FEWFNPLW-------TDDKLH----LL-----M-----  200
PapFuC        ATAIRNYTKDVHFGLYHS-L--FEWFNPLY-------LQDKKG----GF-----S-----  199
RnFucA1       GAAVRK--RNIRYGLYHS-L--FEWFHPLY-------LLDKKN----GL-----K-----  198
ClFuc         GRALRK--RNIRYGLYHS-L--LEWFHPLY-------LLDKKN----NF-----K-----  202
FucA1         GTALRK--RNIRYGLYHS-L--LEWFHPLY-------LLDKKN----GF-----K-----  197
AlfA          AQAVRA--HGMRFGTYYSSL--LDWTFPHL----PIKDYGSFL----LGND---K-----  197
TmaFuc        AKAVRE--AGLRFGVYYSGG--LDWRFTTE----PIRYPEDLS----YIRP---N-----  197
Blon0248      ADAMRD--KGLKFGVYYSSG--LDWHKEPN----MPIL--GDG----EYGP---Q-----  197
Blon0426      ADAMRD--KGLKFGVYYSSG--LDWHKEPN----MPIL--GDG----EYGP---Q-----  197
Fuc35B        VASCRK--YGIKPGIYAST---TANGFLHV----D--------------------NPGL  161
BbAfcB        VASAKK--YGLKVGIYYSP---ADSYMERK----GVWGNNSARVERTIPTLVENDDRAGK  663
BT2192        VDACEE--YGLKAGIYLGP---HDRHEHLS----P------------------------  157
Fuc18         SDACKK--YGIKFAVYLSP---WDRHQA-------------------------------  156
BiAfcB        SESARR--HGLKFGVYLSP---WDRTEESY----G------------------------  143
BT1625        RNACDK--YDMKFGVYLSP---WDRNAECY----G------------------------  175
Fuc39         RNACDK--YDMKFGVYLSP---WDRNAECY----G------------------------  176
BT4136        RKACKK--YGMRFGLYLSP---WDRNAECY----G------------------------  175
AtFUC1        ASAAKE--AGIGLGLYLSP---WDRHEQCY----G------------------------  162
CpAfc2        SEACAK--YNIKFGVYLSP---WDQNSEHY----G------------------------  172
SpGH29        SQAATE--FDMDMGVYLSP---WDAHSPLY----H------------------------  135
Fuc1584       AEACKR--GGINFGIYFSL---IDWHFPQA----YPISSHN-----------------CD  173
AlfC          AEACQK--AGLKFGLYYSQD--LDWHDPNG----GGYKSNDVETAGT---T---WDNSWD  163
Fuc35A        AEACKK--EGIKLGLYYSLG--RDWEDPDV----PTNWP-----VKA---G---RSNTWD  178
Fuc30         ADAFRA--EGMKVMLYYSI---LDTHARLR----PKC----------------------  149
Fuc19A        FEAFRK--EGFMTGAYFSK---PDWHNQDY----WW--DYF---ATP---N---R--NVN  203
AlfB          VAACRE--EDLLPFFYMAT---YDWHTPLY----DD-----------------------  137
BT2970        VKAYND--EGIDVHFYFSV---MDWSNPDY----RY----------------------D  192
Fuc5372       VKEVRK--QGLKLGFYYSL---LDWSHPDY----PNKTRT------------------E  163
TfFuc1        ADECRK--QGIRLHLYYSH---LDWRRDDY----YPLGRTG---KG-------------  172
Mfuc6         LDAMRA--EGLRVGIYFSL---IDWHHPDY----PAFTDAD---RPY---R---WGQ---  154
Mfuc5         VDAFRA--EGLKVGFYYSL---LDWHHPDY----PIAGDSI---HPM---R---N--HPD  147
aLfuk1        ADAFRA--EGLKVGFYYSI---IDWHHPDY----PAYGDRQ---HPM---R---D--NAE  151
Fuc193        VNAFRN--EGIRIGFYYSL---LDWHHPDF----TI--DRN---HPQ---M---PQNPEL  180
Mfuc2         VEAFRA--EGLKIGFYHSL---LDWHHPDF----PV--DGL---HPM---R---D--DLA  151
Mfuc7         ADAFRA--EGLRVGFYYSL---IDWHHPDF----PI--DML---HPR---R---E--DTD  152
Mfuc1         VEAFRA--EGIRVGFYYSI---IDWHHPEF----PV--DVF---HPL---R---D--HPD  151
Mfuc4         VEAFRA--EGLHVGFYYSI---IDWHHPDF----LI--DVF---HPL---R---N--HPE  150
                        .
```

**Fig. S2.** Continues on the next page.

```
FgFCO1        --------GQLPRDEEDQ-LWFDKHREMLDH-----V-QPDIIWNDF---SLDSPGECGS   261
NixE          DDADVANDWTPVSQAWLD-DWLARTTELIDV-----Y-QPDLIYFDW---WIAHPTFR--   319
Fuc2358       DHFDLT-SKPYPSKEFLE-DWLLRTCELVRD-----Y-QPELLYFDW---WIQHESFR--   260
FoFCO1        TNKSIEYTGYVEVNDFINDIQNPQMEALFYD-----Y-DIEMLWCDI---GGPNKAPD--   391
DdFuc         ------------TQVYVDEILMKQLKDIVTT-----Y-EPELIWADG---DWMQLSNY--   231
DmFuca        ------------QQHFVERKVRPEQMELVQQ-----Y-LPEIIWSDG---DWEAPAKY--   237
PapFuC        ------------TQTYTLDICLPELHEIIEN-----Y-KPDVLWSDG---DWEALPEY--   236
RnFucA1       ------------TQHFVSTKTMPELYDLVNR-----Y-KPDLIWSDG---EWECPDSY--   235
ClFuc         ------------TQFFVRAKTMPELYDLVNR-----Y-EPDLIWSDG---EWKCPDTY--   239
FucA1         ------------TQHFVSAKTMPELYDLVNS-----Y-KPDLIWSDG---EWECPDTY--   234
AlfA          ------------SQTYKD-YVWHQWHELIDR-----Y-HPDVLWNDI---GYPDDHR---   232
TmaFuc        ------------TYEYAD-YAYKQVMELVDL-----Y-LPDVLWNDM---GWPEKGKE--   233
Blon0248      ------------SEDYAR-YMYSHVMDLIDE-----Y-QPSILWGDI---DVPKISEE--   233
Blon0426      ------------SEDYAR-YMYSHVMDLIDE-----Y-QPSILWGDI---DVPKISEE--   233
Fuc35B        VKKGS----PVTQEEYNK-IVETQLTELWSN-----YGKLFEIWFDG---GVLSQ-----   203
BbAfcB        VASGKLPTFKYKATDYGA-YMLNQLYELLTE-----YGDISEVWFDG---AQGNT-----   709
BT2192        ---------LYTTERYKE-YYAHQLGELMSD-----YGKIWETWWDG---AGADE-----   194
Fuc18         ---------NYGTPEYVD-YFYKQLHELLTN-----YGDVFEIWFDG---ANGGDGWYGG   198
BiAfcB        ---------K--GKAYDD-FYVGQLTELLTQ-----YGPIFSVWLDG---ANGEG-----   178
BT1625        ---------D--SPKYNE-FFIRQLTELLTN-----YGEVHEVWFDG---ANGEG-----   210
Fuc39         ---------D--SPKYNE-FFIRQLTELLTN-----YGEVHEVWFDG---ANGEG-----   211
BT4136        ---------D--SPRYNK-FFIRQLTELLTN-----YGEVHEVWFDG---ANGEG-----   210
AtFUC1        ---------K--TLEYNE-FYLSQMTELLTK-----YGEIKEVWLDG---AKGDG-----   197
CpAfc2        ---------EGNGGDYNE-FYMNQLRELLTN-----YGPIAEVWMDG---AKGSN-----   209
SpGH29        ---------VDREADYNA-YYLAQLKEILSNPNYGNAGKFAEVWMDG---ARGEG-----   177
Fuc1584       F---ITP-------QHHE-FTKAQVTELLTN-----YGPISELWFDM---GS--------   206
AlfC          F---PDEDQKNFDLCFDN-KILPQIKEIMSN-----YGDIATAWFDV---PM--------   203
Fuc35A        Y---PDEDSKQLPAYIER-KVKPQLKELLTN-----YGEIAVIWFDT---PE--------   218
Fuc30         -----------ITPQHIE-MIKEQLRELLTN-----YGEITALIIDGWDAPWSRIS----   188
Fuc19A        Y---KIERHPEKWEAFKT-YTHNQIGELMSN-----YGTIDILWLDG---GWVSPR----   247
AlfB          -----------DFPAYLT-YLQKSVEVLCRN-----YGPVGGFWFDG---NWNK------   171
BT2970        I---KSKEDSIAFSRFLE-FTDNQLKELATR-----YPTVKDFWFDG---TWDASV----   236
Fuc5372       V---RYKNDPERWARFNK-FNFGQLAELNKT-----W-KPDLYWFDG---DWEQ------   204
TfFuc1        ----TGRTTQGKWEDYCA-FMNNQLTELLTN-----YGLIGAIWFDG---MWDKDI-YP-   217
Mfuc6         ----WRRSSPEAWVRYQQ-FMFGQIRELLTE-----YGKIDIIWFDG---GWER------   195
Mfuc5         F---Q--NHTGNLPRYAD-YVRDQVRELLTN-----YGMIDIIWFDF---SYGEMR-GE-   191
aLfuk1        F---K--DRPQDFNRYLD-YMHGQVKELLTN-----YGTIDVLWFDF---SYEDMT-GE-   195
Fuc193        L---KELNKNRNMAKYRE-YMKNQLTELLTE-----FGQIDELFMDY---TYAE------   222
Mfuc2         F---REAAQGRDMARYRE-YLFGQTRELLTQ-----FGKIDIMWFDF---SYSELN-WG-   197
Mfuc7         A---REMSHGRDIRKYAC-YMRDQVRELLTN-----YGKIDILWFDF---SYGSWT-GEG   199
Mfuc1         V---AEINQGRDVRRYAE-YLRNQVEELLTR-----YN-PEILWCDF---SYPHAT-YK-   196
Mfuc4         V---ARLNEGRDMRRYAA-YMRNQVTELLTN-----YGDIDIIWFDF---SYPGRD-YN-   196
                                         :                 *
```

**Fig. S2.** Continues on the next page.

```
FgFCO1          ------FEGPCAVDEQKRLEFLAYYFNRGE---EWGKEVVTTYKHHD-------------   299
NixE            ---------------SSLPTMLAYYYNQGAARTEADRGVVVNYKLG--------------   350
Fuc2358         ---------------PYLMRFLAYYYNLAA---QEDRKVAVCYKQD--------------   288
FoFCO1          ------------VL----APWLNWARDQGR-------QVTFNDRCGA-------------   415
DdFuc           ------------WK---STEFLSWLYTNSSVKD----TVIVNDRWGSEC----RD-----   263
DmFuca          ------------WR---SEEFIAWLYNDSPVRD----TVVTNDRWGFGT----AC-----   269
PapFuC          ------------WN---STEFLAWLCNDSPVKD----TVVTNDRWGQGT----SC-----   268
RnFucA1         ------------WN---STEFLAWLYNESPVKD----QVVVNDRWGQNC----SC-----   267
ClFuc           ------------WN---STEFLSWLYNDSPVKD----HVVVNDRWGQNC----SC-----   271
FucA1           ------------WN---STNFLSWLYNDSPVKD----EVVVNDRWGQNC----SC-----   266
AlfA            -----------------LETLFKYYYQQVP-------EGLVNDRWQQFP----DWMRTSW   264
TmaFuc          ------------DL----KYLFAYYYNKHP-------EGSVNDRWGV-------------   257
Blon0248        ------------DNDFSVARLFEHYYDVVP-------DGVVNDRWGL-------------   261
Blon0426        ------------DNDFSVARLFEHYYDVVP-------DGVVNDRWGL-------------   261
Fuc35B          -----QNG---------GADILTLIQRLQP-------NSIAFQGPYGYP-NLIRWVGNEE   241
BbAfcB          -------AGTEHYD---YGVFYEMIRRLQP-------QAIQ-ANAA----YDARWVGNED   747
BT2192          ---------LTTPV---YRHWYKIVREKQP-------DCVIFGTKNSYPFADVRWMGNEA   235
Fuc18           AKDARTIDRKTYYD---YPRAYKMIDELQP-------QAVIFSDGG----PGCRWVGNEN   244
BiAfcB          -----KNGKTQYYD---WDRYYNVIRSLQP-------DAVI-SVCG----PDVRWAGNEA   218
BT1625          -----PNGKKQIYD---WDAFYKTIQQLQP-------KAVM-AIMG----DDVRWVGNEK   250
Fuc39           -----PNGKKQIYD---WDAFYKTIQQLQP-------KAVM-AIMG----DDVRWVGNEK   251
BT4136          -----PNGKKQVYD---WDTVYETIHRLQP-------KAVM-AIMG----DDIRWVGNES   250
AtFUC1          -------EKDMEYF---FDTWFSLIHQLQP-------KAVIFSDAG----PDVRWIGDEA   236
CpAfc2          -------V-KQEYN---FEEWFALIKELQP-------ECLIFSPQG----PDIRWIGNEK   247
SpGH29          -------AQKVNYE---FEKWFETIRDLQG-------DCLIFSTEG----TSIRWIGNER   216
Fuc1584         --------N-TPEQ---SKELYQLVHRLQP-------DCMVSGRLGNDQ-----------   236
AlfC            --------TLSEAQ---SQTIYDTVRELQP-------NCLINSRLGNGK-----------   234
Fuc35A          --------LVTRQQ---SKELRELIHSLQP-------GCLINSRIGNGL-----------   249
Fuc30           ---------YDDVP---FEDIYRLVKSIQP-------NCLVMDLNAAKY----PA-----   220
Fuc19A          --------NNQDID---MPQIAAMARAKQP-------GLLVVDRTIHGK-----------   278
AlfB            --------KDADWH---LPELYGMIRHYQP-------NAIIVNNTGLKN----RGQVSD-   208
BT2970          --------KKNGWW---TAHAEQMLKELVP-------GVAINSRLRADD----KGKRHFD   274
Fuc5372         --------SAEAWN---SKDIVDLLRSDNK-------NVIINSRIQGY------------   234
TfFuc1          -----DGMTAKTWN---LNEQYTLIHRLQP-------ACLIGNNHHITP----FA-----   253
Mfuc6           --------TPDEWK---ATELEAMIRSLQP-------EILINDRLPGA------------   225
Mfuc5           -----------AWK---ATELVKMVRELQP-------GIIIDNRLGGDI----KAAEPE-   225
aLfuk1          -----------KWK---ATELVKMIRELQP-------NVLIDNRLGGNI----KAREPE-   229
Fuc193          ---GENGKNSKDWD---AEGIVKLARKLQP-------QIIINNRLGLTE----NR-----   260
Mfuc2           ---WSKGRGKADWH---SEELMAMVRELQP-------GILLNDRLEVG------------   232
Mfuc7           DKAWMKGKGKDDWE---SEELIRIARELQP-------GIIIDNRTEIE------------   237
Mfuc1           ---GLPGKGRNDWQ---SESLVAMIRAISP-------KIILNNRLDLPI----A------   233
Mfuc4           ---GLPGKGRNEWE---SEKLYALVRRLRP-------HIIIDNRLDLPA----HL-----   234
```

**Fig. S2.** Continues on the next page.

```
FgFCO1        -----------------HG-------FRNTSAV------DDWER----------------  313
NixE          ------------------A-------FPEGAGT------LDIER----------------  363
Fuc2358       ------------------A-------LPFGSGI------VEMER----------------  301
FoFCO1        ---------------------------AGDYST------PEYA-----------------  425
DdFuc         --------------------------KNGGFYT------GADH-----------------  274
DmFuca        --------------------------MHGDFYN------CADR-----------------  280
PapFuC        --------------------------KHGGFYS------CNDR-----------------  279
RnFucA1       --------------------------RHGGYYN------CEDK-----------------  278
ClFuc         --------------------------HHGGYYN------CQDK-----------------  282
FucA1         --------------------------HHGGYYN------CEDK-----------------  277
AlfA          IRPIFNLVAAQVIKRDQHHSNDLSEVKYYDYRT------FEYRT----------------  302
TmaFuc        --------------------------PHWDFKT------AEYHV----------------  269
Blon0248      --------------------------THWDFRT------VEYEQ----------------  273
Blon0426      --------------------------THWDFRT------VEYEQ----------------  273
Fuc35B        ------------------------GNSPYPCWATADATTSADGVQKI-------------  264
BbAfcB        ------------------------GWARQTEWSPQAAYNDGVDKV----------SLKPG  773
BT2192        ------------------------GEAGDPCWATTDSVAIRDEAQYY-------------  258
Fuc18         ------------------------GFAGATNWSFLRGGEVYPGYPKY-------------  267
BiAfcB        ------------------------GHVRDNEWSVVPRRLRSAELTMEKSQQEDDASFATT  254
BT1625        ------------------------GLGRETEWSATVLTPG-------IYARSEENNKRLG  279
Fuc39         ------------------------GLGRETEWSATVLTPG-------IYARSEENNKRLG  280
BT4136        ------------------------GLGRETEWSTTVLTPE-------IYARADKNNKKLG  279
AtFUC1        ------------------------GLAGSTCWSLFNRTNAKIGDTEP-------------  259
CpAfc2        ------------------------GYAGEPCWSTIDIEKMK-ERENP-------------  269
SpGH29        ------------------------GYAGDPLWQKVNPDKLG-TEAEL-------------  238
Fuc1584       ----------------------------YDFSV--------MADNTY-------PEGSL-  252
AlfC          ----------------------------YDFVS--------LGDNEI-------PKNKED  251
Fuc35A        ----------------------------GDYSI--------IEQKLS-------NS----  262
Fuc30         -----------------------EALFYTDIKS--------YEQGAG-------QHIS--  240
Fuc19A        ---------------------------YENYQT--------PEQKIP-------EHQ---  293
AlfB          --------------------------PEIDVVT--------YERRTP-------DEIYHG  227
BT2970        ----------------------SNGRLMGDYES-------GYERRLP-------DPVK--  296
Fuc5372       ----------------------------GDYAT--------PEQGVP-------VVRP--  249
TfFuc1        ---------------------------GEDIQI--------FERDLP-------GENKAG  271
Mfuc6         ----------------------------GDYDT--------PEQFVP-------PQLP--  240
Mfuc5         -------------------------IYAGDFAS--------PEQIIP-------PEGV--  243
aLfuk1        -------------------------IYAGDFAS--------PEQLLP-------PHGI--  247
Fuc193        --------------------------QGWDYIT--------PGQFMP-------QQWP--  277
Mfuc2         ----------------------------GDIVT--------PEQYQP-------HGWM--  247
Mfuc7         ----------------------------QDLWT--------PEQYQP-------TEWV--  252
Mfuc1         ----------------------------ADIYT--------PEQVQP-------TEWV--  248
Mfuc4         ----------------------------ADVHT--------PEQWQP-------TEWV--  249
```

**Fig. S2.** Multiple sequence alignment (MSA) of characterized GH29A and GH29B α-L-fucosidases listed in CAZy and α-L-fucosidases previously isolated from the bacterial intestinal metagenome by our group[1], using Clustal Omega[2]. Residue numbering for each sequence is indicated on the right side of the alignment. Nucleophile, acid-base and residues selected for mutations in Fuc2358 and Fuc5372 are highlighted, representing residues that are structural homologs in both enzymes based on AlphaFold2 analysis in the same color (H101 from Fuc5372 and H132 from Fuc2358, orange; W151 from Fuc5372 and F184 from Fuc2358, purple; D199 in Fuc5372 and D251 in Fuc2358, green; R230 in Fuc5372 and K286 in Fuc2358, pink; E241 in Fuc5372 and E300 in Fuc2358, red; Q242 in Fuc5372 and R301 in Fuc2358, blue). Residues sequence homologous to the residues selected for mutations in Fuc2358 and Fuc5372 are framed in the same black box. Residues that are conserved in all sequences in the alignment are indicated with "*" below sequences; conserved substitutions are indicated with ":"; semi-conserved substitutions are represented with ".". FgFCO1: *Fusarium graminearum* AFR68935.1; NixE: *Xanthomonas campestris* pv. *campestris* AAM42160.1; Fuc2358: uncultured *Streptococcus* sp. UUB87428.1; FoFCO1: *Fusarium oxysporum* AFR68934.1; DdFuc: *Dictyostelium discoideum* AAO51149.1; DmFuca: *Drosophila melanogaster* AAM50292.1; PapFuC: *Patiria pectinifera* BBG92283.1; RnFucA1: *Rattus norvegicus* CAA34268.1; ClFuc: *Canis lupus familiaris* CAA63362.1; FucA1: *Homo sapiens* AAA52481.1; AlfA: *Lactobacillus casei* CAQ67115.1; TmaFuc: *Thermotoga maritima* TAAD35394.1; Blon0248*: Bifidobacterium longum* subsp. *infantis* ACJ51376.1; Blon0426: *Bifidobacterium longum* subsp. *infantis* ACJ51546.1; Fuc35B: uncultured *Bacteroides* sp. UUB87424.1; BbAfcB: *Bifidobacterium bifidum* BAH80310.1; BT2192: *Bacteroides thetaiotaomicron* AAO77299.1; Fuc18: uncultured *Bacteroides* sp. UUB87420.1; BiAfcB: *Bifidobacterium longum* subsp. *infantis* ACJ53394.1; BT1625: *Bacteroides thetaiotaomicron* AAO76732.1; Fuc39: uncultured *Bacteroides* sp. UUB87425.1; BT4136: *Bacteroides thetaiotaomicron* AAO79241.1; AtFUC1: *Arabidopsis thaliana* NP_180377.2; CpAfc2: *Clostridium perfringens* ABG83106.1; SpGH29: *Streptococcus pneumoniae* AAK76203.1; Fuc1584; uncultured *Phocaeicola* sp. UUB87427.1; AlfC: *Lactobacillus casei* CAQ67984.1; Fuc35A: uncultured *Bacteroides* sp. UUB87423.1; Fuc30: uncultured *Bacteroides* sp. UUB87422.1; Fuc19A, uncultured *Bacteroides* sp. UUB87421.1; AlfB: *Lactobacillus casei* CAQ67877.1; BT2970: *Bacteroides thetaiotaomicron* AAO78076.1; Fuc5372: uncultured *Phocaeicola* sp. UUB87429.1; TfFuc1: *Tannerella forsythia* AEW21393.1; Mfuc6:

uncultured bacterium AIC77303.1; Mfuc5: uncultured bacterium AIC77302.1; ALfuk1: *Paenibacillus thiaminolyticus* CBM40947.1; Fuc193: uncultured *Bacteroides* sp. UUB87426.1; Mfuc2: uncultured bacterium AIC77299.1; Mfuc7: uncultured bacterium AIC77304.1; Mfuc1: uncultured bacterium AIC77298.1; Mfuc4: uncultured bacterium AIC77301.1.

**Supplementary material references**


1. Moya-Gonzálvez EM, Peña-Gil N, Rubio-del-Campo A, *et al*. Infant gut microbial metagenome mining of α-L-fucosidases with activity on fucosylated human milk oligosaccharides and glycoconjugates. *Microbiol Spectr.* 2022;10(4):e0177522. doi:10.1128/spectrum.01775-22

2. Sievers F, Wilm A, Dineen D, *et al.* Fast, scalable generation of high-quality protein multiple sequence alignments using Clustal Omega. *Mol Syst Biol*. 2011;7:539. doi:10.1038/msb.2011.75
